# Kynurenine importation by SLC7A11 propagates anti-ferroptotic signaling

**DOI:** 10.1016/j.molcel.2022.02.007

**Published:** 2022-03-03

**Authors:** Alessandra Fiore, Leonie Zeitler, Marion Russier, Annette Groß, Maria-Kathrin Hiller, Joanne L. Parker, Luca Stier, Thomas Köcher, Simon Newstead, Peter J. Murray

**Affiliations:** 1 Max Planck Institute for Biochemistry 82152 Martinsried, Germany; 2 Department of Biochemistry, University of Oxford Oxford, United Kingdom; 3 Vienna BioCenter Core Facilities GmbH Vienna, Austria

## Abstract

IDO1 oxidizes tryptophan (TRP) to generate kynurenine (KYN), the substrate for 1-carbon and NAD metabolism and is implicated in pro-cancer pathophysiology and infection biology. However, the mechanistic relationships between IDO1 in amino acid depletion versus product generation have remained a long-standing mystery. We found an unrecognized link between IDO1 and cell survival mediated by KYN that serves as the source for molecules that inhibit ferroptotic cell death. We show this effect requires KYN export from IDO1-expressing cells, which is then available for non-IDO1-expressing cells via SLC7A11, the central transporter involved in ferroptosis suppression. Whether inside the “producer” IDO1^+^ cell or the “receiver” cell, KYN is converted into downstream metabolites, suppressing ferroptosis by ROS scavenging and activating an NRF2-dependent, AHR-independent cell protective pathway, including SLC7A11, propagating anti-ferroptotic signaling. IDO1 therefore controls a multi-pronged protection pathway from ferroptotic cell death, underscoring the need to re-evaluate the use of IDO1 inhibitors in cancer.

## Introduction

In mammals, tryptophan (TRP) is sourced exclusively from the diet and microbial flora. Beyond its essential role in protein biosynthesis, mammalian TRP metabolism is compartmentalized in an organ- and context dependent way by two main pathways (Fiore and Murray, 2021). First, TRP is the precursor of the neuromodulators serotonin and melatonin (and their derivatives), and the anti-ferroptotic molecule indole-3-pyruvate (Zeitler et al., 2021). These biochemical pathways leave the indole ring intact. The second pathway involves TDO2, which is predominantly expressed in the liver and responsible for most of the dietary tryptophan metabolism, and IDO1. Both enzymes break the indole ring to form kynurenine which is the precursor for many important biochemical pathways including de novo NAD biosynthesis (Minhas et al., 2019) and 1-carbon metabolism (Newman et al., 2021). IDO1 is inducible by interferons and in some cells constitutively expressed, especially in many tumors (Meireson et al., 2020; Uyttenhove et al., 2003). Another IDO family member, IDO2, is encoded by a gene adjacent to IDO1. However, the precise enzymatic or biological function of this isoform remains controversial (Austin et al., 2010).

The first clues about the relationship between IDO1 and TRP metabolism were made with scant knowledge of the existence of the enzyme, which had yet to be cloned. In these studies, intracellular proliferation of pathogens such as *Chlaymdia sp*. and *Toxoplasma gondii* was suppressed by IFNγ in a TRP-dependent way, giving rise to the idea that intracellular pathogens scavenged host TRP and that a defense against pathogen amino acid scavenging might be a host regulated pathway to suppress TRP availability (Byrne et al., 1986; Pfefferkorn, 1984; Pfefferkorn et al., 1986). Subsequently, IDO1 was identified as the IFNγ-inducible enzyme that destroyed TRP. However, despite decades of work, the precise mechanistic relationship between IDO1, TRP, intracellular pathogen growth and survival remains unclear. Similar to the situation inside a Toxoplasma-infected cell, tumor microenvironments (TME) have limiting amounts of TRP, and this depletion is thought to be mediated by constitutive and regulated IDO1 (and possible TDO2) expression in infiltrating myeloid cells and tumor cells themselves, combined with the anabolic requirements of the tumor mass (Murray, 2016). As all cells require TRP, the net depletion within the TME conceivably blocks the proliferation of anti-tumor immune cells, such as T cells or any TRP-dependent immune cells and also their effector functions (Mellor and Munn, 1999; Murray, 2016). Ergo, the principle behind developing IDO1 inhibitors was two-fold; first to block IDO1 in tumor cells, which may be dependent on KYN production for 1-carbon units, NAD biosynthesis and other products of TRP, including KYN itself, which can bind to and activate the aryl hydrocarbon receptor (AHR) (Schmidt and Bradfield, 1996) and second, by inhibiting IDO1 activity in tumor-infiltrating myeloid cells. The combined cell-specific effects of IDO1 inhibition would alter the metabolic milieu of the TME to, for example, restoring free TRP for T cell proliferation.

The conversion of TRP to KYN in any inflammatory setting raises several unanswered questions that have remained open for over four decades. First, it is still unknown how IDO1^+^ cells manage their TRP metabolism. In theory, once a cell expresses IDO1, the flux through the KYN pathway increases, requiring TRP import via yet to be identified TRP transporters. Second, cells in the neighborhood of IDO1^+^ cells (and the IDO1^+^ cells themselves) will be exposed to a TRP-deficient microenvironment, potentially rich in KYN. The complexity of the TRP-depletion versus metabolite generation remains a puzzle of cellular biochemistry with a key practical implication: IDO1 inhibitors have thus far been unsuccessful in cancer therapy, despite IDO1 being one of the most rational and tractable pathways to target (Long et al., 2019).

Here, we use genetic and cellular biochemical approaches to dissect TRP depletion versus TRP metabolite-mediated pathways. We uncover an unrecognized link between IDO1 and cell survival mediated by KYN that serves as the source for molecules that suppress ferroptotic cell death. We show this effect requires three interacting mechanisms: first, KYN is exported from IDO1^+^ cells and available to be imported by non-IDO1-expressing cells via SLC transporters including SLC7A11, the central transporter involved in ferroptosis suppression required for cystine import. Whether inside the “producer” IDO1^+^ cell or the “receiver” cell, KYN is then converted to downstream metabolites of which 3-hydroxy-kynurenine (3HK) and 3-hydroxyanthranilic acid (HAA) have potent ROS-scavenging anti-ferroptotic activity. Finally, KYN activates an NRF2-dependent, AHR-independent cell protective pathway, and SLC7A11 expression, further increasing KYN import to propagate anti-ferroptotic signaling. IDO1 therefore controls a multi-pronged anti-ferroptotic death pathway.

## Results

### IDO1-driven TRP metabolism protects against ferroptosis

**Figure 1:**
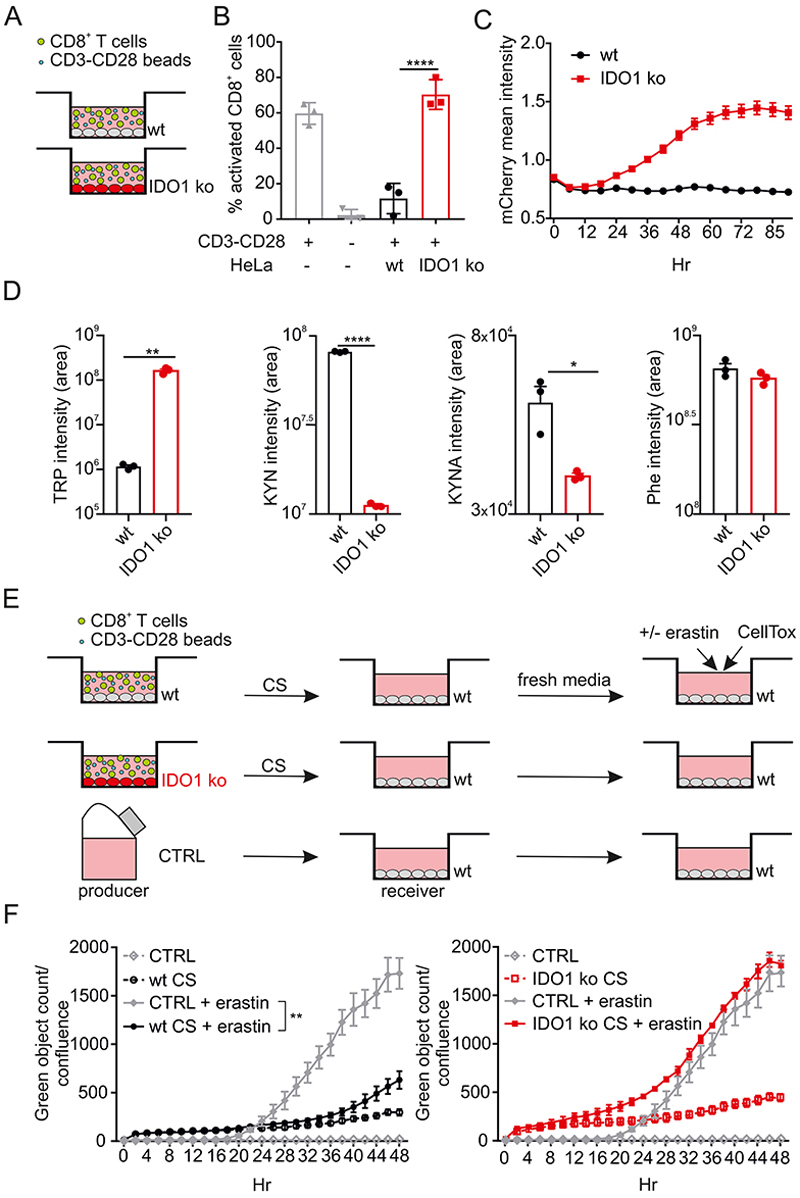
IDO1 induces T cells proliferation arrest and resistance to ferroptosis. (A) Simplified schema of the co-culture between mitomycin-treated wt or IDO1 ko HeLa cells with allogeneic CD8^+^ lymphocytes in the presence of CD3-CD28 dynabeads. (B) Quantification of CD8^+^ T cell proliferation, using CFSE staining, in the presence of wt or IDO1 ko HeLa cells relative to CD8^+^ T cell control. (C) Quantification of IDO1 expression by tracking mCherry by live imaging during the co-culture with CD8^+^ T cells. (D) Intensity area values for tryptophan (TRP), kynurenine (KYN), kynurenic acid (KYNA) and phenylalanine (Phe) detected by LC-MS-based targeted metabolomics in the supernatant after 48 hr of co-culture between wt or IDO1 ko HeLa cells with CD8^+^ lymphocytes. (E) Schematic depicting the experimental approach used in F. CS, conditioned supernatant. (F) 24 hours of pre-conditioning of wt HeLa cells with the supernatant from the co-cultures with wt, but not IDO1 ko HeLa cells, protects from erastin-induced ferroptosis. Cell death was monitored over time using CellTox, counting for green objects normalized to cell confluence. B, C, D and F n=3 biological replicates, bars are SDs *p < 0.05, **p < 0.01, ****p < 0.0001 for multiple comparisons calculated using one-way ANOVA with Tukey’s HSD test in (B) and (F) and two-tailed Student’s t test for pairwise comparisons in (D).

A central concept behind IDO1 inhibitor development for cancer centered on the TRP-dependent cross-talk in the TME between IDO1-expressing tumor, myeloid cells, and T cells whose proliferation would be suppressed by local TRP restriction. Therefore, we first aimed to mimic an IDO1-dependent TME where we could assess how tumor and CD8^+^ T cells interacted when TRP metabolism was manipulated through IDO1 expression. We generated a reporter cell line for IDO1 using HeLa cells, where mCherry was inserted precisely at the position of the start codon of the IDO1 gene, creating a complete IDO1-deficiency (Figure S1A and S1B). In these cells, mCherry reporter expression paralleled the normal expression of IDO1 in response to IFNγ, allowing visualization of IDO1 expression kinetics (Figure S1C and S1D). When we cultured mitomycin-treated wt HeLa cells (as a “bystander” tumor cell model) with activated human CD8^+^ T cells, T cell proliferation was arrested; by contrast, loss of IDO1 in the bystander HeLa cells restored T cell proliferation (Figure 1A and 1B). In this co-culture system, IDO1 expression was dependent on activated CD8^+^ T cells, which produce IFNγ to drive IDO1 expression at the mRNA and protein levels, leading to proliferative arrest (Video S1 and Figure 1C). As we anticipated, after 48 hr of co-culture with human CD8^+^ T cells, induced IDO1 expression in the wt HeLa cells caused depletion of TRP in the culture media with corresponding production of KYN and kynurenic acid (KYNA), the two main metabolites of IDO1-TRP catabolism. We observed the opposite effect in the IDO1-deficient cultures, while metabolism of phenylalanine (Phe), another aromatic amino acid, was equivalent in both cell systems (Figure 1D, Table S1). The intracellular amounts of KYN and KYNA, as well as the levels of more downstream metabolites of the KYN pathway (KP), such as HAA, NADH and NAD^+^ were also increased, consistent with flux along the TRP metabolic pathway (Figure S1E, Table S1).

At this point, we had a tractable IDO1-dependent experimental system to evaluate the outcomes of TRP depletion versus metabolite generation. Given that KYN was secreted into the medium, we first considered that this metabolite and its derivatives could modulate tumor cell proliferation and survival in an autocrine-paracrine way, as well as influencing neighboring cells. As indole-3-pyruvate from IL4i1, another TRP metabolizing enzyme that leaves the indole ring intact, has potent anti-ferroptotic properties (Zeitler et al., 2021), we wondered if IDO1 metabolites had similar cell protective effects. To assess this, we set up a co-culture between CD8^+^ T cells, and wt or IDO1 ko cells for 48 hr, as described before. We transferred conditioned supernatant (CS) from these cultures to untreated HeLa cells. After 24 hr we changed the medium and challenged the cells with the ferroptosis inducer, erastin, which blocks cystine uptake through SLC7A11 and thereby disrupts glutathione homeostasis (Figure 1E). Cells incubated with the CS from the co-culture with wt HeLa cells were completely protected from erastin-induced ferroptosis (Figure 1F). By contrast, the conditioned media derived from the IDO1 ko co-culture had no protective effects.

To confirm this finding in a different way, we used conditioned media from wt HeLa cells treated or not for 48 hr with IFNγ and we removed IFNγ (IFNγ homodimer ~ 40 kDa) and any other components >10 kDa by size filtration (Figure S1F). The results of these experiments revealed that soluble factors <10 kDa and dependent on IDO1, protected against erastin- and RSL3-induced ferroptosis (Dixon et al., 2012) (Figure S1G). Most importantly, IDO1 ko HeLa cells were also protected, suggesting that IDO1 is not required in the “receiving” cell as long as the “producer” cell expressed IDO1 (Figure S1H).

A key question in clinical cancer therapy concerns the thus far reported poor or negligible efficacy of IDO1 inhibitors (IDO1i) (Eynde et al., 2020). However, links between IDO1 and ferroptosis, which may have key roles in cancer cell survival have yet to be recognized. Thus, we asked if IDO1i reversed IDO1-dependent anti-ferroptotic signaling. We used a similar approach as described above; HeLa cells were stimulated for 48 hr with IFNγ to induce IDO1 with or without three frontline IDO1i: epacadostat (INCB024360), BMS986205 (linrodostat) and indoximod (NLG-8189). Analysis of cell death at 48 hr showed that none of the inhibitors had a direct anti-ferroptotic effect (Figure S2A). To discriminate between the IFNγ and IDO1-mediated effects on ferroptosis regulation (Wang et al., 2019), we again used size-dependent filtration to remove the cytokine and then the CS was used to treat wt or IDO1 ko HeLa cells. After 24 hr, the cells were challenged with erastin. However, epacadostat and BMS986205 (not indoximod, discussed further below) reversed the IDO1-dependent ferroptosis protection (Figure S2B, C). Consistent with the CS transfer results above (Figure S1H), IDO1 ko HeLa cells had an identical death response as the wt cells, confirming that a factor in the media was IDO1-dependent, and was transferable, independent on IDO expression in the receiver cell (Figure S2D).

### IDO1-metabolism induces a broad stress and redox-protective gene expression program

**Figure 2:**
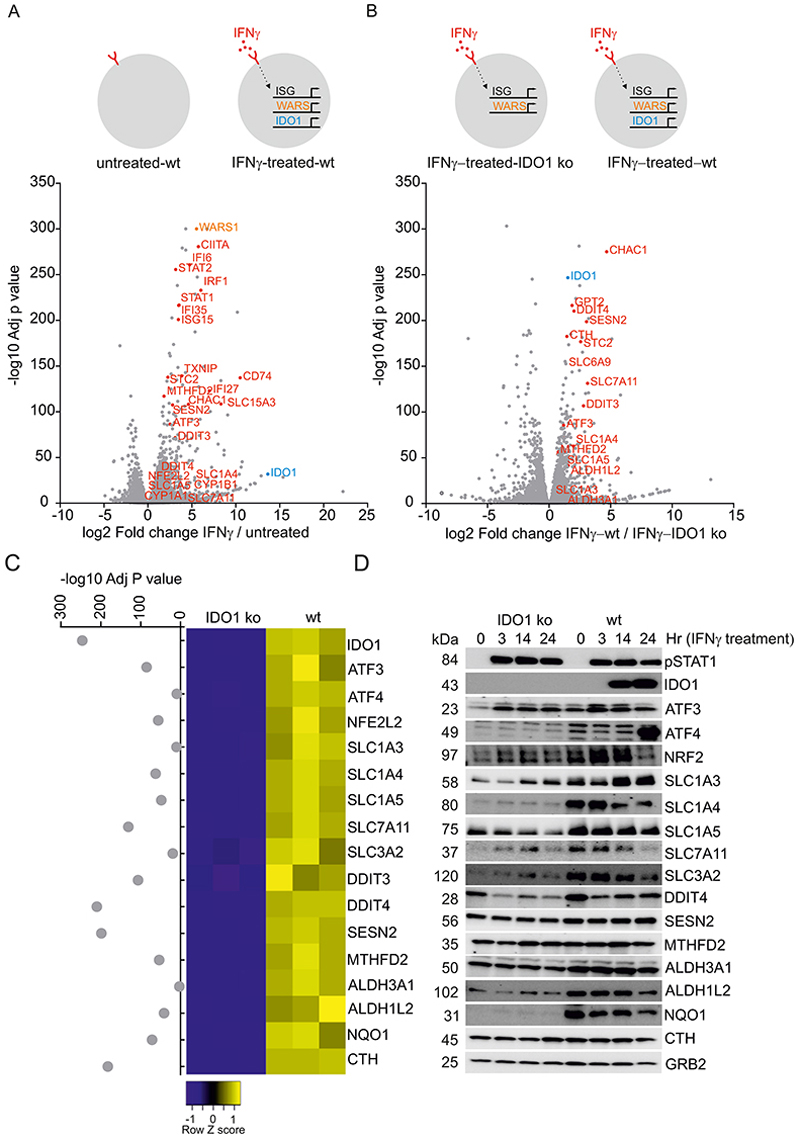
Dissection of IDO1-dependent gene expression (A, B) Schematic depicting the experimental approach and volcano plots showing overall changes in the transcriptome in untreated versus IFNγ-treated wt HeLa cells (A) and in wt versus IDO1 ko cells (B). (C) Heatmap of selected genes that were significantly differentially expressed after 24 hr of IFNγ treatment in wt versus IDO1 ko HeLa cells (adjusted p-value <0.05). (D) Immunoblot validation of selected target proteins from (C) after 3, 14 and 24 hr of of IFNγ treatment. (A-C) Log-adjusted p value was calculated from three independent biological replicates

To understand the molecular links between IDO1 and anti-ferroptotic signaling, we performed RNAseq analysis on wt HeLa cells treated with IFNγ versus untreated HeLa cells as a control. Besides IDO1, which is one of the most significantly upregulated mRNAs (Log_2_ fold change=13.64; adjusted p-value p=1.37E-32), IFNγ stimulates the expression of more than 2000 transcripts (adjusted p-value <0.05), many of which encode antiviral proteins (Figure 2A). Our matched IDO1-deficient cells provided an opportunity to separate the IFNγ-dependent anti-viral signature from the IDO1-dependent effects on the transcriptome, all within in the context of IFNγ signaling (Figure 2B). This analysis revealed IDO1 was required to control a suite of mRNAs linked to cellular and oxidative stress responses, including solute carriers (SLC) SLC1A3, SLC1A4, SLC1A5, SLC3A2 and SLC7A11, transcription factors ATF3, ATF4, NRF2 and several downstream target mRNAs such as DDIT4, DDIT3, SESN2, NQO1, ALDH1L2 and ALDH3A1 (Figure 2C). We found IDO1 controlled the expression of many mRNAs encoding enzymes involved in 1-carbon metabolism including MTHFD2 and CTH, supporting the recent finding that TRP-derived carbons enter the purine nucleotide biosynthesis (Newman et al., 2021). Moreover, protein validation of these targets revealed IDO1-dependent transcriptional changes did not always correlate with protein abundance, suggesting IDO1 controls a complex regulatory network at the transcriptional and translational levels (Figure 2D). We further confirmed those observations by comparing the transcriptome of IFNγ-treated wt and IDO1 ko HeLa cells with cells concurrently treated with epacadostat (1µM) and BMS986205 (1µM), which completely abrogated the IDO1-dependent cellular stress gene expression with near identical overlap to the IDO1 ko cells, arguing these drugs are highly specific for IDO1. By contrast, indoximod (100 µM) had a far weaker effect on suppressing the IDO1 pathway (Figure S2E and S2F). Therefore, we defined epacadostat and BMS986205 as true on-target IDO1 inhibitors.

### KYN-derived metabolites suppress ferroptosis

**Figure 3:**
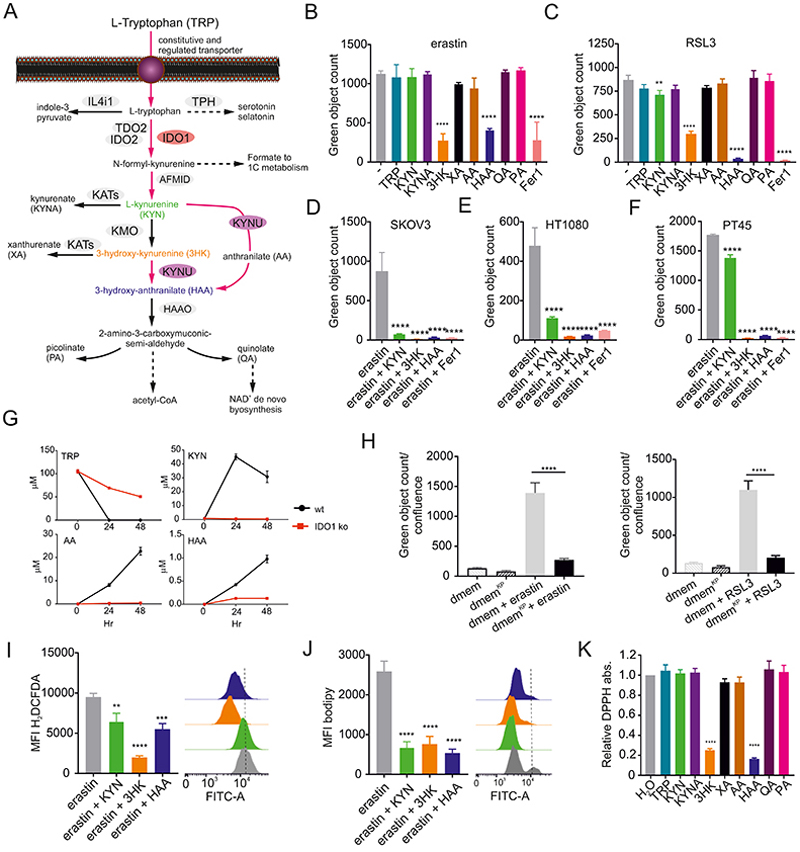
KYN metabolites protect against ferroptosis. (A) Simplified diagram of the TRP catabolism pathway. (B, C) Quantification of cell death in HeLa cells treated for 48 hr with 10 µM of erastin (B) or 1 µM of RSL3 (C) in the presence of 200 µM of TRP, KYN, KYNA, 3HK, xanthurenic acid (XA), anthranilic acid (AA), HAA, quinolate (QA), and picolinate (PA). 2 µM of Fer-1 was used as a control to block ferroptosis. (D, E, F) Quantification of cell death in SKOV3 (D), HT1080 (E) and PT45 (F) cells concurrently treated for 48 hr with erastin and 200 µM KYN, 3HK and HAA. Fer-1 was added as a control. (G) MS-based quantitative metabolomics of supernatant of wt vs. IDO1 ko HeLa cells after 24 and 48 hr IFNγ treatment. (H) Quantification of cell death in HeLa cells treated for 48 hr with 10 µM of erastin (left) 1 µM of RSL3 (right) in the presence or not of a physiologically relevant media (dmem^KP^ standing for DMEM reconstituted with “KYN Pathway” metabolites) that contains 30 µM KYN, 22 µM AA and 1 µM HAA. (I) KYN, 3HK and HAA block ROS accumulation induced by 8 hours of erastin treatment. ROS quantification was determined by flow cytometry using the H_2_DCFDA probe. (J) KYN, 3HK and HAA block lipid peroxidation induced by 8 hr of erastin treatment. Lipid peroxidation was determined by flow cytometry using the fluorescent lipid peroxidation reporter molecule, C11-BODIPY. (K) Cell-free scavenging activity of 200 µM of each metabolite determined by changes in the absorbance at 517 nm of the stable radical DPPH relative to water control (n = 4 technical replicates, bars are SDs). B, C, D, E, F, H, I, J and K n= 3 biological replicates, bars are SDs *p < 0.05, **p < 0.01, ***p < 0.001, ****p < 0.0001 for multiple comparisons calculated using one-way ANOVA with Dunnett’s test (B, C, D, E, F, I, J and K) and with Tukey’s HSD test in (H).

Activation of IDO1 expression in the TME or in intracellular pathogen infection leads to a depletion of TRP and the corresponding production of KYN. The relative biological role of these effects remains unresolved, mainly because of the challenge of accounting for one effect versus the other across time (Fiore and Murray, 2021). In the context of ferroptosis protection, we next devised experiments to quantify the effects of TRP starvation versus product generation. We first assessed the direct anti-ferroptotic effects of KYN and its downstream metabolites (Figure 3A). We treated HeLa cells (without IFNγ-mediated IDO1 induction) with 200 µM of KYN and a range of KYN-derived metabolites with erastin or RSL3 to induce ferroptosis. As expected, cell death was not reversed by the pan-caspase inhibitor z-VAD-FMK or Necrostatin1S (Nec1S) while Ferrostatin-1 (Fer-1), N-acetylcysteine (NAC) and Liproxtatin-1 (Lipr1) were all protective, confirming ferroptosis was the mode of death in this system (Dixon et al., 2012; Kelly et al., 2021; Tang et al., 2021)(Figure S3B). KYN metabolites 3HK and HAA potently blocked ferroptosis, causing maintenance of cell viability throughout 48 hr of exposure to ferroptosis inducers, while all other IDO1-derived metabolites had weak or no protective effects. KYN itself showed an intermediate phenotype and delayed the onset of ferroptosis (Figure S3A, 3B and 3C). None of the KYN-derived metabolites affected the growth rate or viability of the cells under normal culture conditions (Figure S3C). We titrated the concentration of 3HK or HAA and found both active in a concentration range starting from ~12.5 µM (Figure S3D and S3E). We further verified these observations with multiple cell lines: SKOV3 cells, an ovarian cancer cell line that constitutively expresses IDO1 (Figure 3D), HT1080 cells, a human fibrosarcoma cell line frequently used in ferroptosis studies (Figure 3E) and PT45 cells, an aggressive pancreatic adenocarcinoma epithelial cell line (Figure 3F). The effect of the metabolites was further confirmed by inducing ferroptosis without synthetic chemical manipulation by cysteine (CYS) starvation (Poltorack and Dixon, 2021). CYS deprivation triggered ferroptotic death with a similar kinetic to cells treated with erastin and these starved cells were completely protected by the concurrent treatment with KYN, 3HK and HAA (Figure S3F and S3G). These results were further confirmed by quantifying cell death using 7-AAD and Annexin-V staining by flow cytometry and by inducing cell death with RSL3 and erastin treatments (Figure S3H, I).

To verify if the concentration range of the metabolites were within a physiologically relevant range, we performed mass spectrometry-based quantitative metabolomics in the supernatant of wt and IDO1 ko HeLa cells 24 and 48 hr after activation of IDO1 expression by IFNγ treatment. In the supernatant from wt control HeLa cells, we could not detect any tryptophan after 24 hr of IDO1 induction as expected; by contrast, in the same samples, we detected 45 ± 2 µM of KYN; this concentration is consistent with previous observation of KYN concentrations at ~37 μM in a tumor model (Opitz et al., 2011) (Figure 3G, Table S1). Based on this analysis, we “recreated” the conditioned supernatant with the metabolites at the exact concentration that we detected after 48 hr of IDO1 activity (30 µM KYN, 22 µM AA and 1 µM HAA). This reconstituted media (DMEM^KP^ for KYN pathway media) protected the cells against both erastin- and RSL3-mediated ferroptosis, confirming our previous observations and conclusions that IDO1 mediated the creation of an extracellular anti-ferroptotic milieu (Figure 3H).

**Figure 4:**
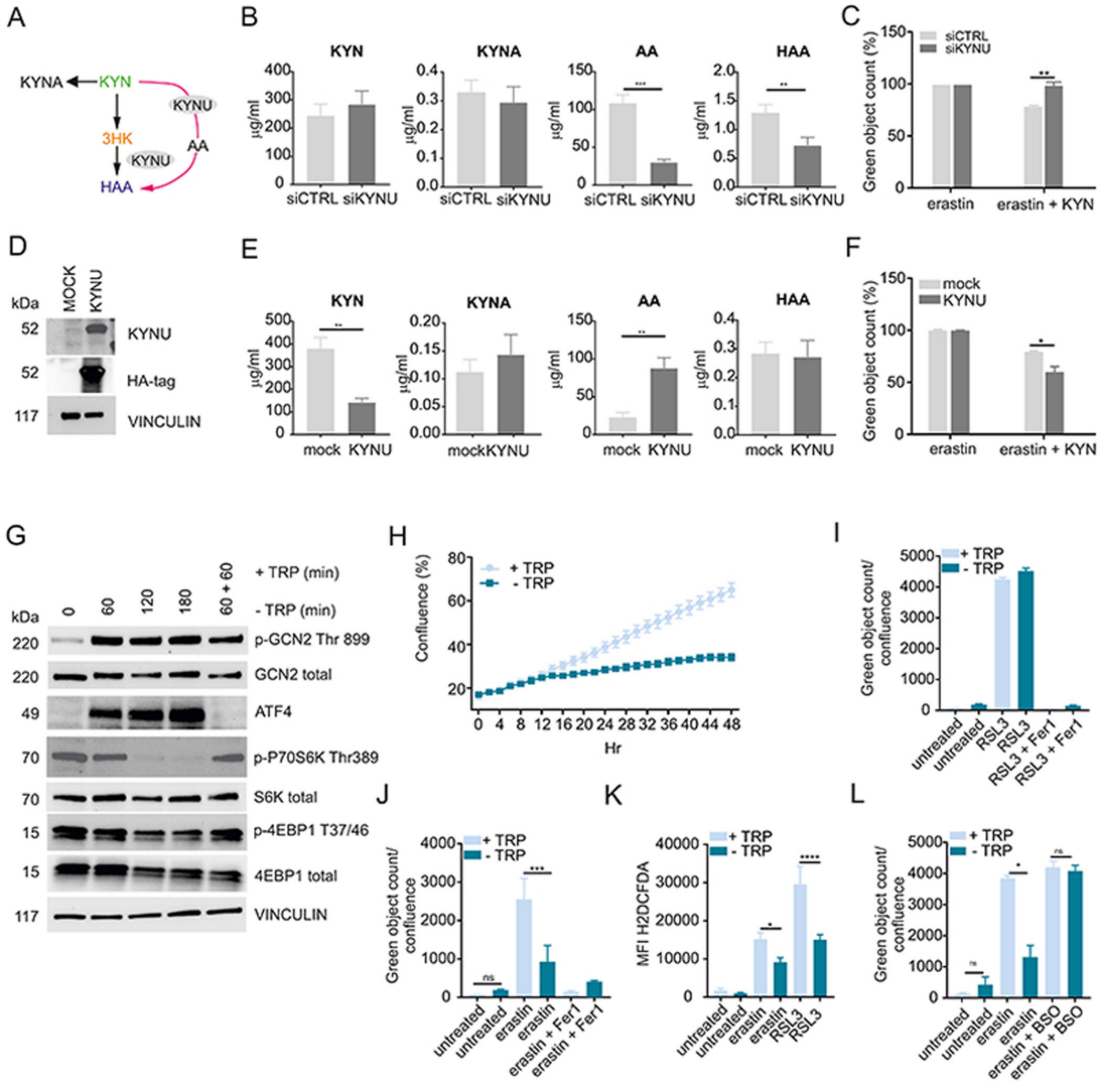
KYN metabolites and TRP starvation protect against ferroptosis. (A) Simplified diagram of the KYNU pathway. (B) MS-based quantitative metabolomics of cell extract of HeLa cells transfected with with KYNU siRNA (siCTRL as negative control) after 24 hr of KYN treatment. (C) Quantification of cell death in siCTRL - or siKYNU-transfected cells treated for 48 hr with 10 µM of erastin in the presence of 200 µM of KYN. Cell death was monitored after 48 hr using CellTox, counting for green objects and expressed as percentage of cell death to the control. (D) Expression of KYNU and HA-tag in HeLa cells 48hr after transfection with a mock or HA-KYNU-plasmid. (E) MS-based quantitative metabolomics of cell extract of HeLa cells transfected with with HA-KYNU (a mock plasmid was used as negative control) after 24 hr of KYN treatment. (F) Quantification of cell death in mock- or HA-KYNU-transfected cells treated for 48 hr with 10 µM of erastin in the presence of 200 µM of KYN. Cell death was monitored after 48 hr using CellTox, counting for green objects and expressed as percentage of cell death to the control. (G) Immunoblotting analysis of mTOR activity and ISR over time during TRP deprivation in HeLa cells. (H) Over time quantification of cell growth of HeLa cells in the presence or absence of TRP. (I,J) Quantification of cell death in HeLa cells treated for 48 hr with 10 µM of RSL3 (I) or erastin (J) in the presence of rich (+TRP) or TRP deprived media (-TRP). Fer-1 was added as a control. Cell death was monitored after 48 hr using CellTox, counting for green objects and, where indicated, normalized to cell confluence. (K) TRP deprivation blocks ROS accumulation induced by erastin and RSL3 determined by flow cytometry using H_2_DCFDA probe after 8 hr of treatment. (L) Quantification of cell death in HeLa cells treated for 48 hr with 10 µM of erastin in the presence of rich (+TRP) or TRP deprived media (-TRP) and in the presence of 1 mM of BSO. B, C, E, F, H, I, J, K and L n= 3 biological replicates, bars are SDs *p < 0.05, **p < 0.01, ***p < 0.001, ****p < 0.0001 unpaired t-test for B and E and one-way ANOVA with Tukey’s HSD test in (C, F, I, J, K, L).

We then tested the mechanism(s) of ferroptosis protection mediated by IDO1-derived metabolites. As KYN, 3HK and HAA suppressed intracellular ROS and lipid peroxidation after erastin treatment (Figure 3I and 3J), we suspected each compound may scavenge free radicals. We quantified the radical scavenging activity of each metabolite at 200 µM using the radical diphenyl-2-picrylhydrazyl (DPPH). Both 3HK and HAA were radical scavengers (Figure 3K) at 6.25 and 12.5 µM, respectively, while KYN had no scavenging properties (Figure S3J). Based on the fact that KYN partially protected against ferroptosis (Figure 3B, C) and partly decreased ROS compared to the more potent effects of 3HK or HAA, we suspected that part of the reason why KYN had anti-ferroptotic properties was related to it being the source of 3HK and HAA via L-Kynurenine hydrolase (KYNU), which catalyzes the cleavage of KYN into AA and 3-HK into HAA (Figure 4A). To test this concept, we suppressed KYNU via siRNAs. KYNU knock-down, significantly decreased the concentration of AA and HAA without affecting the amount of KYNA (Figure 4B, Table S1). In this setting, the reduced activity of KYNU resulted in the loss of ferroptosis protection (Figure 4C). By contrast, over-expression of KYNU (Figure 4D) caused an increased in KYN consumption with increased AA production compared to the control cultures (Figure 4E, Table S1). The forced metabolism of KYN via KYNU resulted in protection against ferroptosis (Figure 4F). Collectively, IDO1 expression generates KYN, which is subsequently used to make 3HK and, via KYNU, HAA. 3HK and HAA are potent free radical scavengers, in part accounting for the anti-ferroptotic effects of IDO1.

### TRP restriction suppresses ferroptosis

The preceding experiments established that KYN and its metabolites were anti-ferroptotic in part via their ROS scavenging properties. However, the question of the contribution of TRP depletion to ferroptosis protection remained open. Therefore, we next tested the contribution of TRP starvation to modulate ferroptosis relative to the effect of IDO1-derived metabolites. TRP deprivation, via the activity of IDO1, triggers the amino acid integrated stress response (ISR) which activates the GCN2/ATF4 pathway and inhibits mTOR (Figure 4G) with consequent inhibition of cell proliferation (Figure 4H). The effect of IDO1-dependent metabolites can be separated from tryptophan deprivation by the use of TRP-free media. RSL3-induced ferroptosis was not suppressed in cells cultured in TRP-deficient media compared to conventional DMEM (Figure 4I). However, when we induced ferroptosis by erastin in TRP-free media, death was suppressed and coupled to a lower accumulation of ROS (Figure 4J, K). The explanation for this difference lies in the mode of ferroptosis induction: RSL3 irreversibly inhibits GPX4, preventing the glutathione-dependent detoxification of lipid peroxides, which leads to cell death after ~2-4 hr of treatment. By contrast, erastin blocks the uptake of CYS, which decreases the intracellular reservoir of glutathione (GSH) over a longer time window. Under normal growth and nutrient conditions, protein synthesis is thought to take precedence over other CYS-consuming pathways (Yu and Long, 2016). In theory, therefore, when we block proliferation and translation by TRP deprivation, CYS consumption via the translational machinery declines, allowing an increase in the pool CYS available to generate GSH. Consistent with this idea, the protective effect of TRP starvation was blunted when we co-treated the cells with erastin and buthionine sulfoximine (BSO) that targets the rate-limiting enzyme of GSH biosynthesis, γ-glutamylcysteine synthetase (γ-GCS), indicating that the de novo GSH synthesis is required for the anti-ferroptotic effect of TRP starvation (Figure 4L).

### KYN metabolites protect against ferroptosis independent of the AHR but dependent of NRF2

Our transcriptome analysis (Figure 2) that identified IDO1-mediated but IFNγ-independent gene transcription showed a time-dependent induction of a range of gene and protein expression linked to ISR, a network of cell adaptive changes elicited under different stresses, including amino acid stress (Costa-Mattioli and Walter, 2020). In this context, TRP starvation activates the GCN2 ISR kinase and subsequently depends on transcription factors such as ATF4, ATF3 and NRF2. The combined activity of these transcription factors in turn leads to adaptive changes in amino acid transporter expression, glutathione and 1-carbon metabolism, and other cell protective pathways (Ben-Sahra et al., 2013; Torrence et al., 2021). In addition, KYN binds to the aryl-hydrocarbon receptor (AHR), causing translocation and activation of a complex gene expression pathway tied to stress protection and cell fate decisions (Bessede et al., 2014; Opitz et al., 2011).

**Figure 5:**
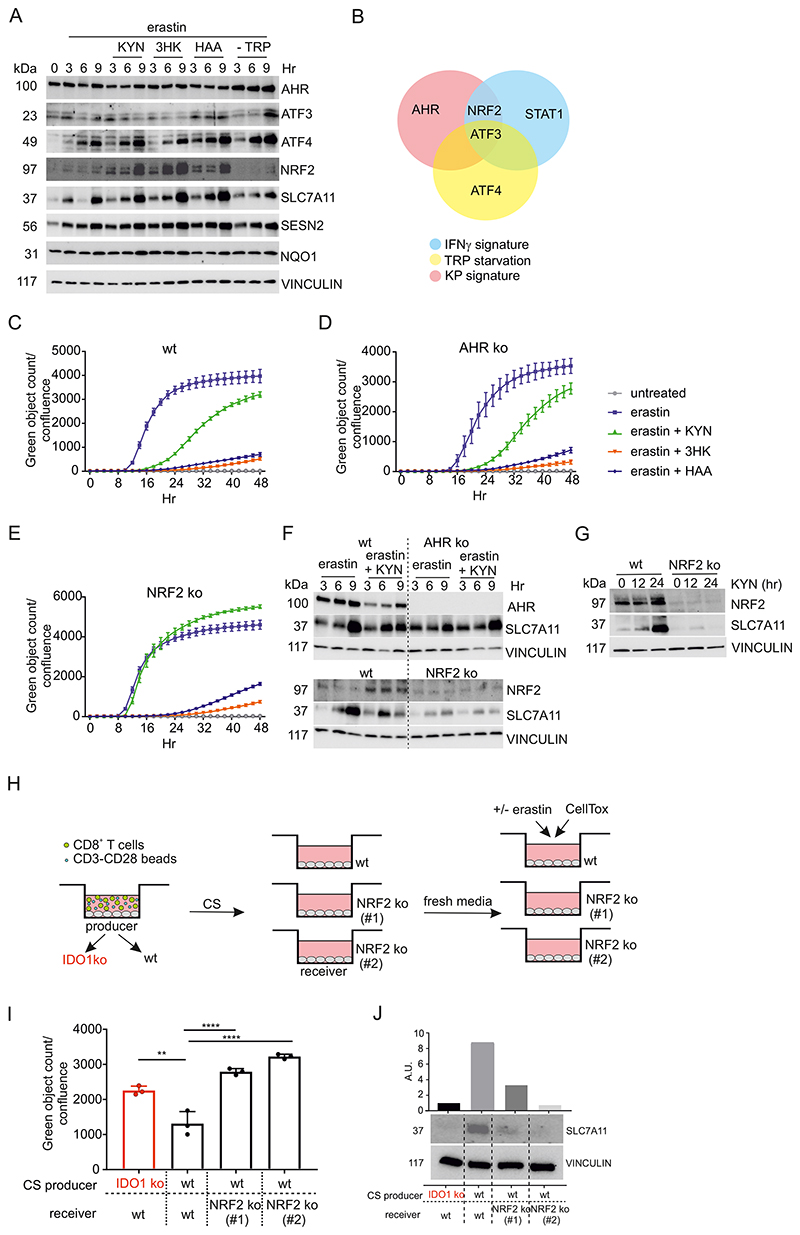
KYN metabolites protect against ferroptosis through the NRF2/SLC7A11 axis (A) Immunoblot analysis of HeLa cells concurrently treated for 3, 6 and 9 hr with erastin and 200 µM KYN, 3HK, HAA or TRP starved. (B) Venn diagram summarizing the contribution of each component of IDO1 activity in the redox-protective program. (C - E) Wt (C), AHR ko (D) or NRF2 ko (E) HeLa cells were concurrently treated for 48 hr with erastin in the presence of 200 µM KYN, 3HK and HAA. Cell death was monitored over time using CellTox, counting for green objects normalized to cell confluence. n = 3 biological replicates, bars are SDs. (F) Loss of NRF2, but not AHR, prevents KYN-dependent SLC7A11 upregulation. Immunoblot analysis of wt, AHR ko and NRF2 ko HeLa cells concurrently treated for 3, 6 and 9 hr with erastin and 200 µM KYN. (G) Immunoblot analysis of NRF2 and SLC7A11 in wt or NRF2 ko HeLa cells treated for 12 and 24 hr with 200 µM of KYN. (H) Schematic depicting the experimental approach used in H and I. CS, conditioned supernatant. (I) Quantification of erastin-induced cell death in wt or NRF2 ko HeLa cells pre-conditioned for 24 hr with supernatant from 48 hr of the co-colture between wt or IDO1 ko HeLa cells with allogeneic CD8^+^ lymphocytes in the presence of CD3-CD28 dynabeads. Cell death was monitored at 48 hr using CellTox, counting for green objects normalized to cell confluence. (J) Immunoblot analysis and quantification of the protein blots relative to the vinculin loading control of SLC7A11 in wt and NRF2 ko HeLa cells treated for 24 hr with the supernatant from the co-colture between wt or IDO1 ko HeLa cells with CD8+ lymphocytes in the presence of CD3-CD28 dynabeads. n = 3 biological replicates, bars are SDs. **p < 0.01, ****p < 0.0001 for multiple comparisons calculated using one-way ANOVA with with Tukey’s HSD test.

We quantified the expression of key ISR transcription factors in response to TRP deprivation or addition of the KP metabolites (in normal media with TRP) under oxidative stress caused by erastin-treatment. TRP deprivation strongly induced ATF3 and ATF4 consistent with the adaptive starvation response but not NRF2 expression, which, by contrast, was strongly induced by the KP metabolites. Strikingly, among the target genes, SLC7A11 was upregulated in all conditions (Figure 5A and 5B). We generated AHR- or NRF2-deficient HeLa cells (Figure S4A and S4B) and treated them with KYN, 3HK or HAA in the presence of erastin to induce ferroptosis. 3HK and HAA completely protected all genotypes against erastin-induced cell death, which we attribute the retained protective effect to their ROS scavenging properties (Figure 3K). However, KYN retains its protective effect in the AHR-deficient cells but not in the NRF2 ko cells (Figure 5C, 5D and 5E). We confirmed these findings using a second knockout clone for each target protein (Figure S4C). In addition, we observed that the erastin- and KYN-dependent SLC7A11 upregulation was reduced in the NRF2 ko (Figure 5F-G) while the AHR ko cells, which showed an intact NRF2 up-regulation (Figure S4D), displayed SLC7A11-expression comparable to the WT cells. Thus, anti-ferroptosis via the KP is partly dependent on NRF2, but independent of the AHR.

We further evaluated the effect of NRF2 on ferroptosis without the addition of exogenous metabolites by utilizing the IDO1-dependent anti-ferroptotic CS (Figure 1). Using the co-culture system described before (Figure 1E), we transferred CS to untreated wt or two independently-generated NRF2 ko HeLa cell clones. CS from an IDO1 ko co-culture was also transferred to wt HeLa cells as a control. After 24 hr we changed the medium in all conditions and then challenged the cells with erastin to induce ferroptosis (Figure 5H). The conditioned media derived from the wt co-culture protected against ferroptosis compared to the CS derived from IDO1 ko culture. By contrast, the CS derived from the wt co-culture had no protective effects on the NRF2 ko cells (Figure 5I). Moreover, when we transferred the same CS used for the ferroptosis assays to wt or NRF2 ko cells for 24 hr, SLC7A11 was up-regulated only when WT CS was transferred to wt cells (Figure 5J). Similar results were obtained using CS derived from WT HeLa cells treated or not with IFNγ to induce IDO1 activity (analogous experiment to Figure S2B, Figure S4E-G). We concluded that KYN induces the NRF2-SLC7A11 axis as anti-ferroptotic defense and this pathway works in parallel with ROS scavenging by KYN metabolites.

### SLC7A11 mediates KYN transport in cancer cells

**Figure 6:**
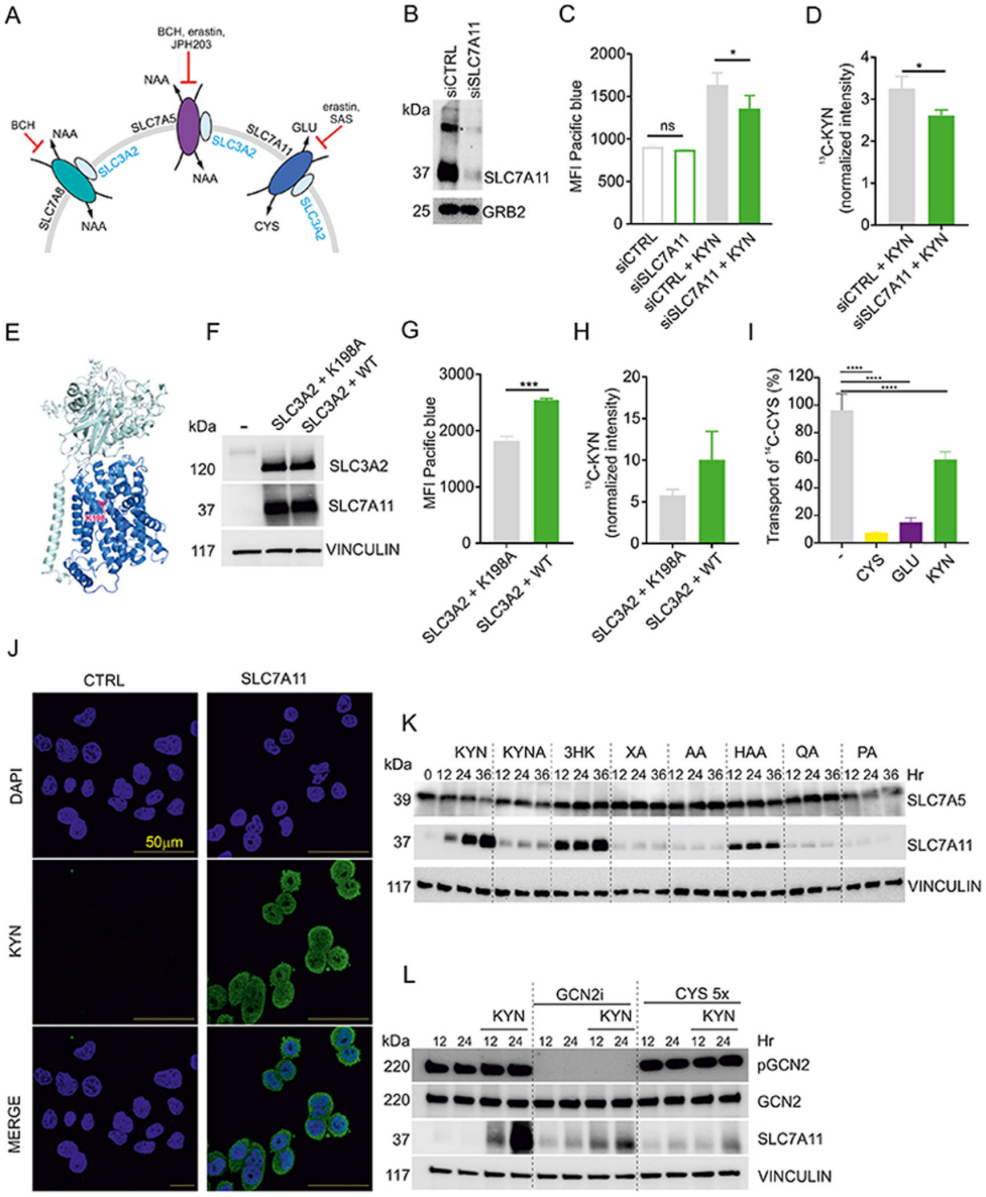
SLC7A11 mediates KYN transport in cancer cells (A) Simplified scheme of SLC7 transporters and relative inhibitors. (B) SLC7A11 immunoblot of HeLa cells transfected with SLC7A11 siRNA (siCTRL as negative control). (C) Flow cytometry evaluation of KYN uptake upon 5 min of treatment with 200 µM of KYN in transfected cells in B. KYN uptake was monitored by flow cytometry analyzing the mean fluorescence intensity (MFI) in the Pacific blue channel (one of three independent transfection experiments is shown, n = 3 biological replicates, bars are SDs). (D) Quantification of total D6-KYN uptake after 5 min of treatment with 200 µM of KYN into transfected cells in B (n = 3 biological replicates, bars are SDs). (E) Molecular structure representation of the complex SLC7A11/SLC3A2 showing the K198A mutation. (F) Immunoblot of HeLa cells transfected with SLC3A2 and SLC7A11 wt or K198A-overexpressing plasmids (an empty vector was used as a negative control). (G) Flow cytometry evaluation of KYN uptake upon 5 min of treatment with 200 µM of KYN in transfected cells. KYN uptake was monitored by flow cytometry analyzing the mean fluorescence intensity (MFI) in the Pacific blue channel (one of three independent transfection experiments is shown, n = 3 biological replicates, bars are SDs). (H) Quantification of total D6-KYN uptake after 5 min of treatment with 200 µM of KYN into transfected cells in F (n = 3 biological replicates, bars are SDs). (I) Transport assays of ^14^C-cystine (^14^C-CYS) uptake for cells transfected with plasmids containing wild type SLC7A11 and SLC3A2 in the presence of 1 mM competing non-radioactive substrates. (n> 6 biological replicates, bars are SDs). (J) Immunofluorescence of KYN accumulation in CTRL cells and HeLa cells transfected with SLC7A11 after 15 min of treatment with and 200 µM KYN (representative images of one of three independent transfection experiments). (K) Immunoblot analysis of SLC7A5 and SLC7A11 in HeLa cells treated for 12, 24 and 36 hr with 200 µM of KYN, KYNA, 3HK, XA, AA, HAA, QA, and PA. (L) Immunoblot of HeLa cells concurrently treated with 200 µM KYN for 12 or 24 hr and 500 nM of GCN2i or 5 times more cystine (CYS) compared to normal medium. *p < 0.05, **p < 0.01, ***p < 0.001, ****p < 0.0001 for multiple comparisons calculated using one-way ANOVA with Tukey’s HSD test in (C) and Dunnett’s test (I) and unpaired t-test for D, G and H.

SLC7A11 (and the related amino acid transporter SLC7A5) is the target of erastin, which blocks cystine transport into cells via an unknown mechanism, which disrupts cysteine and therefore, glutathione homeostasis (Dixon et al., 2012). SLC7A11 mediates cystine import coincident with glutamate export (Dixon et al., 2014). SLC7A11 belongs to the L-type amino acid SLC7 transporter family and forms a heterodimeric amino acid transporter by interacting with the glycoprotein CD98 (SLC3A2) through a conserved disulfide; SLC3A2 also complexes with SLC7A5 and SLC7A8 (Figure 6A) (Mastroberardino et al., 1998), which bear strong sequence identity (Figure S5A). SLC7A5 transports KYN into activated T cells (Sinclair et al., 2018) and given the similarity between SLC7A5 and SLC7A11, we wondered if there was a connection between KYN transport and SLC7A11. This would link several components of the IDO1 anti-ferroptotic pathway as exported KYN could be imported by bystander cells and converted into 3HK and HAA even if such cells did not express IDO1, or stimulate the NRF2 pathway as shown above, enforcing SLC7A11 expression and ferroptosis protection.

Deletion of SLC7A11 renders many tissue culture-adapted cells, including HeLa cells, unable to proliferate *in vitro* (Arensman et al., 2019). We therefore used siRNAs to transiently knockdown SLC7A11 expression (Figure 6B). Due to the fluorescent properties of KYN, we monitored KYN uptake by flow cytometry once KYN accumulated inside the cells (Sinclair et al., 2018). An important caveat in these experiments is that HeLa cells constitutively express SLC7A5, a known KYN transporter. However, KYN fluorescence was decreased in the SLC7A11-knockdown HeLa cells compared to the control after 5 min of exposure with 200 µM of KYN (Figure 6C). D6-metabolite flux analysis (D6-KYN), which we used to quantify intracellular accumulation of KYN, confirmed KYN-fluorescence results (Figure 6D, Table S1). We extended these findings by using the a physiologically relevant concentration of KYN (45 µM of KYN, Figure S5B,C, Table S1) and four additional cancer cell lines, SKOV3, HT1080, PT45 and U2OS that showed varying baseline expression of SLC7A11 (Figure S5D-N).

Next we used selective inhibitors that blocks SLC7A5 (JPH203) (Okunushi et al., 2020), SLC7A11 (sulfasalazine, SAS) (Nagane et al., 2018) or multiple transporters at the same time (erastin and BCH) (Figure 6A). In all conditions, we observed a lower KYN accumulation consistent with the notion that both SLC7A5 and SLC7A11 transport KYN (Figure S5J). To test the opposite scenario by increasing the expression, we transiently overexpressed SLC3A2 in combination with WT or a K198A transport dead mutant SLC7A11. The K198A mutation results in an inactive transporter without affecting cellular location or overall structural integrity as determined by Cryo-EM (Parker et al., 2021) (Figure 6E, F). KYN uptake was increased relative to control transfections (Figure 6G, H). We confirmed this finding by overexpressing SLC7A11(Fig. S5 K, L). Under these conditions, cystine transport was partly blocked by KYN, noting that the target cells have constitutive expression of SLC7A5 (Figure 6I). We also used immunofluorescence with a highly specific anti-KYN antibody (Yamamoto et al., 2019) to show rapid KYN accumulation in the cytoplasm at 5 and 15 minutes following KYN addition (Figure 6J and S6A). Taken together, these results implicate SLC7A11 in mediating KYN transport, in addition to its role as a cystine transporter.

A remaining question is How do cells balance the importation of cystine in the presence of a KYN-rich microenvironment? In our cellular system, cells upregulate SLC7A11 (but not SLC7A5) when exposed to KYN, 3HK and HAA (Figure 6K) resulting in an increased uptake of cystine (Figure S6B). By contrast, KYN importation can be competitively blocked using excess of CYS (Figure S6C-D, Table S1). These data prompted us to speculate that in the presence of a KYN-rich microenvironment, transient competition could occur between KYN and cystine through SLC7A11. Results from T cell biology indicate that cells react to mTOR inhibition by activating signals of “pseudo-starvation” (Procaccini et al., 2021); in our system KYN hinders cystine uptake thus triggering the activation of the ISR through GCN2, increase of ATF4 translation and promotion of SLC7A11 expression for cystine acquisition (Fig. S6E). We corrupted this axis by using a GCN2 inhibitor (GCN2i) (Nakamura et al., 2018) or by restoring the balance between KYN and cystine by increasing the concentration of exogenous CYS (Figure 6L). Therefore, our results argue that the combined action of SLC7A5 and SLC7A11 allows KYN importation produced from IDO1^+^ cells (in an autocrine-paracrine way). KYN catabolism thereby generates 3HK and HAA to provide immediate ROS scavenging and activates an NRF2-dependent gene expression resulting further cystine and KYN uptake by SLC7A11 up-regulation and ferroptosis protection across a wide time frame.

## Discussion

Our results define previously unknown links between IDO1 activity and the suppression of ferroptosis. Research on IDO1 activity in cancer has focused predominantly on the concept that TRP depletion is the main regulatory node of T cell suppression, which spurred the development of IDO1 inhibitors and their testing in clinical cancer settings (Labadie et al., 2019). Our research instead identified the elicitation of anti-ferroptosis pathways as a key consequence of IDO1 activity. We demonstrated KYN generated from TRP can be exported from IDO1^+^ cells and transfer ferroptosis suppression to non-IDO1 expressing cells. We speculate that nodes of IDO1^+^ cells, either tumor cells themselves, or infiltrating myeloid cells, can propagate ferroptosis suppression within the TME. Given that some forms of chemotherapy trigger ferroptosis (Wu et al., 2020), IDO1 may have multiple effects in controlling the outcomes of initial tumor growth by collectively suppressing T cell proliferation and ferroptosis. Most significantly, we were able to link the IDO1 pathway with SLC7A11 at multiple levels, a critical checkpoint in the suppression of ferroptosis required to maintain cysteine and glutathione homeostasis.

Through the use of IDO1-deficient cells, TRP free media and dissection of the products of TRP metabolism downstream of the indole ring-breaking reaction, we were able to assign the contribution of IDO1 to different steps in ferroptosis protection. First, IDO1 promotes the metabolism of TRP to KYN and then, to 3HK and, via KYNU, to HAA; these two downstream metabolites have potent ROS scavenging activity. Thus, a cell that produces or imports KYN and the KYN metabolites have a repertoire of ROS scavengers that work in concert with the anti-oxidative responses. We speculate that in tumors expressing high level of the KP enzymes, specific inhibitors may be useful to corrupt the KYN-derived ROS scavengers. Second, we observed that IDO1 elicits a TRP starvation response identical to the amino acid ISR that enhances a variety of cell protective pathways including the expression of amino acid solute carriers (including SLC7A11), adaptations in 1-carbon metabolism, and enhanced glutathione production, which suppresses ferroptosis. The elucidation of this pathway required a genetic approach, whereby we could define the TRP starvation response in the background of IFNγ signaling. Third, we found that SLC7A11 transported KYN, providing an additional anti-ferroptotic mechanism; in this situation, cells not expressing IDO1 can in theory encounter a TRP-deficient microenvironment, increase SLC7A11 via the ISR and then import KYN to convert to 3HK and HAA. SLC7A11’s close cousin, SLC7A5, was previously shown to import KYN in T cells (Sinclair et al., 2018), suggesting the SLC3A2-dependent amino acid transporter family has a more promiscuous substrate range than previously described. Fourth, in the presence of a KYN-rich microenvironment, competition between KYN and cystine through SLC7A11 is sensed as transient CYS deprivation or “pseudo-starvation”, with consequent activation of the GCN2-ATF4 signaling axis and promotion of SLC7A11 transcription and CYS uptake. Since SLC7A11, SLC7A5 and SLC3A2 are all prominent targets of the ISR, perhaps they form a conserved network to suppress ferroptotic death by multiple approaches.

KYN has previously been shown to bind to and activate the AHR, providing another important link to the pro-tumor effects of IDO1 (Bessede et al., 2014; Liu et al., 2018). Through the generation and use of AHR-deficient cells, we found the AHR was dispensable for ferroptosis protection. Instead, NRF2 played a central role in ferroptosis protection, in part by controlling the expression of SLC7A11. However, 3HK and HAA suppressed erastin or RSL3-induced ferroptosis independent of the AHR or NRF2, highlighting the likelihood that ROS scavenging is the most immediate and proximal way of blocking ferroptosis, while the complex NRF2-dependent pathway controls later adaptations to further suppress death. Conceivably in solid tumors, ROS scavenging and transcriptional and translational adaptations for death protection could occur dynamically across time and spatially, depending on where IDO1^+^ and/ or KYNU^+^ cells reside, the expression level of the amino acids and metabolites transporters, how many of those cells there are and, most importantly how much TRP and CYS is available.

Although the reasons why the numerous IDO1i trials failed to show efficacy remain unknown, our experiments revealed new information about these drugs. First, through the use of a comparative approach, we found that epacadostat and BMS986205 inhibited IDO1 in the context of cellular activity and in fact mirrored the genetic IDO1-deficiency at the transcriptional level. By contrast, indoximod had a limited effect on the IDO1-dependent transcriptome. Epacadostat and BMS986205 have favorable inhibitory and pharmacological properties compared to indoximod and our studies support the notion that the former are highly on-target compounds, while the latter is far less active by comparison (Gunther et al., 2019). Taken in context with the notion that anti-ferroptosis is a component of cancer cell survival (Hangauer et al., 2017), we noted that scant information is available about IDO1 expression in individual patient’s tumors. Without detailed patient-specific information about the stratification of IDO1 expression in the TME, on-target activity of each drug cannot be evaluated. Nevertheless, our results suggest that the active IDO1i, if used in IDO1^+^ tumors, or tumors infiltrated with IDO1^+^ myeloid cells, would corrupt both the anti-ferroptosis pathway uncovered here, and the TRP starvation pathway.

### Limitations of the study

Our results impinge on the concept of “spreading” ferroptosis, a phenomenon observed in different biological contexts including kidney tubule death, and transmission of lipid peroxidation from one cell to another (Davidson and Wood, 2020; Linkermann et al., 2014; Stockwell et al., 2017). We show that anti-ferroptosis can also propagate from one cell to another via KYN, so long as one cell is IDO1^+^ and can generate KYN for export by neighboring cells. In the context of chronic inflammation, such as the TME, the balance of competing pro-and anti-ferroptotic pathways may contribute to the survival of cancer cells under stress. However, this idea highlights a limitation of our work, and indeed most work on amino acid metabolism *in vivo*, namely the current inability to quantify metabolites across time and space in living animals. Although some tumor types are deficient in TRP and arginine, key amino acids involved in immunosuppression and immunoregulation, such studies only provide a snapshot of what occurred inside a tumor at one-time (Murray, 2016; Sullivan et al., 2019). Dynamic imaging approaches will be essential to track amino acid amounts inside tumors as well as the “deorphanization” of the plasma membrane transporters responsible for the import and export of the KP metabolites that can represent valuable alternative targets to inhibit IDO1^+^ tumor cells or tumor-infiltrating myeloid cells. A second limitation is closely related to the above; in vivo biomarker-based detection of ferroptosis is currently lacking. An important gap in knowledge concerns the frequency and rate of ferroptosis relative to other forms of death that manifest inside a tumor. Given the dissimilarity between KYN and cystine, how does a cystine specific transport system recognize KYN? The structure of L-glutamate-bound SLC7A11 highlighted the importance of two arginine side chains (Parker et al. 2021). It is possible that KYN recognition occurs following an interaction between the ring system of the 2-aminophenyl group in KYN and Arg135 on TM3 of SLC7A11. Thus, precise structural information of the SLC3A2-dependent transporters in the act of transporting their different substrates including KYN and cystine – and whether other factors may also be involved - will be essential to designing new ways to manipulate metabolic pathways of cell death.

## Star Methods

### Key Resources Table

**Table:** 

REAGENT or RESOURCE	SOURCE	IDENTIFIER
Antibodies		
IDO1 (D5J4E™) Rabbit mAb	Cell Signaling Technology	86630S
EAAT1 (D44E2) XP® Rabbit mAb	Cell Signaling Technology	5684S
SLC1A4 Antibody	Cell Signaling Technology	8442S
ASCT2 (D7C12) Rabbit mAb	Cell Signaling Technology	8057S
xCT/SLC7A11 (D2M7A) Rabbit mAb	Cell Signaling Technology	12691
AhR (D5S6H) Rabbit mAb	Cell Signaling Technology	83200S
LAT1 Antibody	Cell Signaling Technology	5347S
4F2hc/CD98 (D6O3P) Rabbit mAb	Cell Signaling Technology	13180S
NRF2 (D1Z9C) XP® Rabbit mAb	Cell Signaling Technology	12721S
Phospho-Stat1 (Tyr701) (D4A7) Rabbit	Cell Signaling Technology	7649s
Vinculin (E1E9V) XP® Rabbit mAb	Cell Signaling Technology	13901S
ATF-3 (D2Y5W) Rabbit mAb	Cell Signaling Technology	33593S
4F2hc/CD98 (D6O3P) Rabbit mAb	Cell Signaling Technology	13180S
HA-Tag (C29F4) Rabbit mAb	Cell Signaling Technology	3724S
Sestrin 2 Polyclonal antibody	Proteintech	10795-1-AP
ALDH1L2 Polyclonal antibody	Proteintech	21391-1-AP
ALDH3A1 Polyclonal antibody	Proteintech	15578-AP1
MTHFD2 Polyclonal antibody	Proteintech	12270-1-AP
REDD1 specific Polyclonal antibody	Proteintech	10638-1-AP
Gamma Cystathionase (CTH) Polyclonal antibody	Proteintech	12217-1-AP
NQO1 Polyclonal antibody	Proteintech	11451-AP1
Purified Mouse Anti-GRB2 Clone 81/GRB2	BD	610112
Anti-CREB-2/ATF-4 Antibody (B-3)	Santa Cruz Biotechnology	SC-390063
L-KYN antibody	ImmuSmol	IS003
Anti-L-KYN Hydrolase (KYNU) antibody	Abcam	ab236980
phospho-4EBP1 (T37/46)	Cell Signaling Technology	2855
4EBP1	Cell Signaling Technology	9644
phospho-S6K (T389)	Cell Signaling Technology	9234
S6K	Cell Signaling Technology	2708
Phospho-GCN2 (T899)	Abcam	ab75836
GCN2	Cell Signaling Technology	3302
Annexin-V	BD Biosciences	550474
7-Amino-Actinomycin D (7-AAD)	BD Biosciences	559925
Peroxidase-AffiniPure Goat Anti-Rabbit IgG (H+L)	Jackson ImmunoResearch	111-035-003
Peroxidase-AffiniPure Goat Anti-Mouse IgG (H+L)	Jackson ImmunoResearch	115-035-003
Goat anti-Mouse IgG (H+L) Alexa Fluor 488	Thermo Fisher Scientific	A-11029
Biological Samples		
Human CD8^+^ cells	Vincenzo Bronte lab (University of Verona)	N/A
Chemicals, Peptides, and Recombinant Proteins		
DMEM high glucose, pyruvate	Gibco/Life Technologies	41966052
Dulbecco’s MEM (DMEM) Low Glucose, w/o Amino Acids, Pyruvic Acid (Powder)	Us Biological	D9800-13
Opti-MEM media	Thermo Fisher Scientific	31985062
FBS Superior	Sigma-Aldrich	S0615
Penicillin-Streptomycin	Sigma-Aldrich	P4333
Dynabeads Human T-Activator CD3/CD28	Thermo Fisher Scientific	111.61D
Erastin	Selleckchem	S7242
(1S, 3R)-RSL3	Selleckchem	S8155
Ferrostatin-1	Sigma-Aldrich	SML0583
GCN2-IN-6	MedChemExpress	HY-130240
N-acetylcysteine (NAC)	Sigma-Aldrich	A9165
Z-VAD-FMK	Promega	G7231
Liproxtatin	Selleckchem	S7699
Necrostatin1S	Cell Signaling Technology	17802
Buthionine sulfoximine (BSO)	Sigma Aldrich	B2515
CellTox Green	Promega	G8731
Recombinant Human IFN-gamma Protein	R&D	285-IF
Epacadostat	Cayman chemicals	19875
BMS986205	Chemgood	C-1360
Indoximod (1-Methyl-D-tryptophan)	Sigma-Aldrich	452483
L-tryptophan, CELLPURE® ≥99 %	Carl Roth	1739.1
L-kynurenine reagent grade, ≥98%	Sigma-Aldrich	K8625
3-Hydroxy-DL-kynurenine (CAS 2147-61- 7)	Santa Cruz Biotechnology	sc-21413
Kynurenic acid reagent grade, ≥98%	Sigma-Aldrich	K3375
Xanthurenic acid reagent grade, ≥96%	Sigma-Aldrich	D120804
Anthranilic acid reagent grade, ≥98%	Sigma-Aldrich	A89855
3-Hydroxyanthranilic Acid	TCI	A0316
2-picolinic acid, reagent plus(r), 99%	Sigma-Aldrich	P42800
Quinolinic acid	Bio Techne	0225
L-KYN SULFATE (RING-D4, 3,3-D2, 97%+)	Buchem	
Sulfasalazine, 97%	ACROS Organics™	461240250
JPH203	Selleckchem	S8667
2-amino-2-norbornanecarboxylic acid (BCH)	Sigma-Aldrich	A7902
1,1 -diphenyl-2-picrylhydrazyl (DPPH)	Sigma-Aldrich	D9132
Lipofectamine 3000 Transfection reagent	Invitrogen	L3000008
Lipofectamine RNAi max	Invitrogen	13778150
SuperSignal™ West Pico PLUS Chemiluminescent Substrate	Thermo Fisher Scientific	34580
Halt Protease % Phosphate Single Use Inhibitor Cocktail (100X) Kit	Thermo Fisher Scientific	78444
RIPA buffer	Abcam	ab156034
4–15% Criterion™ TGX Stain-Free™ Protein Gel, 26 well, 15 μl	Biorad	5678085
4–15% Criterion™ TGX Stain-Free™ Protein Gel, 18 well, 30 ul	Biorad	5678084
0.2μM nitrocellulose membrane	Amersham	N010600001
Bovine Serum Albumin (BSA)	Sigma-Aldrich	A2153
Powdered milk	Carl Roth	T145.2
DAPI	Sigma-Aldrich	D9542
Amicon-Ultra 10 kDa columns	Merck Millipore	UFC501024
Mitomycin C	Sigma-Aldrich	M4287
μ-Slide 8 Well Glass Bottom coverslips	ibidi	80827
MG132	Millipore	474790
Critical Commercial Assays		
CellTrace™ CFSE Cell Proliferation Kit, for flow cytometry	Invitrogen	C1157
H2DCFDA (H2-DCF, DCF)	Thermo Fisher Scientific	D399
BioTracker™ Cystine-FITC Live Cell	Merck	SCT047
Deposited Data		
RNAseq Data	This study	GSE174116
Experimental Models: cell lines		
HeLa	ATCC	CCL-2
SK-OV-3	ATCC	HTB-77
U-2 OS	ATCC	HTB-96
HT-1080	ATCC	CCL-121
PT45	Vincenzo Bronte lab (University of Verona)	N/A
Experimental Models: Organisms/Strains		
N/A		
Oligonucelotides		
ON-TARGETplus Human KYNU siRNA	Horizon Discovery	L-009867-00- 0005
SLC7A11 siRNA	Invitrogen	4390824, s24289
Scrambled siRNA	Invitrogen	4390843
Recombinant DNA		
pSpCas9(BB)-2A-GFP (PX458)	Addgene	48138
pEx-K248-mCherry repair plasmid	Eurofins Genomic	N/A
xCT (SLC7A11) Human Tagged ORF Clone	Origene	NM_014331
pcDNA3.1 hygro/KYNU 3xHA	This study	N/A
SLC7A11_pLexM	S. Newstead laboratory (University of Oxford)	N/A
K198A_SLC7A11_pLexM	S. Newstead laboratory (University of Oxford)	N/A
SLC3A2_pLexM	S. Newstead laboratory (University of Oxford)	N/A
Software and Algorithms		
GraphPad Prism	GraphPad software	Version 7
FlowJo	FlowJo	Version 10
Image J	Fiji	N/A
CorelDRAW Graphics Suite 2020	CorelDRAW	Version 2020
IncuCyte Live Cell Analysis System	Sartorius	S3
PyMOL	pymol.org	N/A

### Resource Availability

#### Lead contact

Further information and requests for resources and reagents should be directed to and will be fulfilled by the Lead contact Prof. Peter J. Murray (murray@biochem.mpg.de)

#### Materials Availability

Reagents are available on request to the lead contact.

### Experimental Models And Subject Details

#### Cell culture

HeLa, SK-OV-3, U-2 OS and HT-1080 cell lines were purchased from the American Type Culture Collection (ATCC). PT45 cell line was a gift from Prof. Vincenzo Bronte (University of Verona). All cell lines were tested to be free from Mycoplasma contamination by routine PCR screening. All cell lines were grown in DMEM high glucose, pyruvate (Gibco/Life Technologies Corp.) plus 10% fetal bovine serum (FBS superior, Sigma-Aldrich) and 1% Penicillin-Streptomycin (Sigma-Aldrich). Medium without TRP was prepared using D9800-13 Dulbecco’s MEM (DMEM) Low Glucose, w/o Amino Acids, Pyruvic Acid (Powder, USBiological). All cell lines were grown in humidified tissue culture incubators at 37°C with 5% CO2.

### Method Details

#### CRISPR/Cas9

For the construction of the IDO1 reporter cell line three partly overlapping small guide RNAs (sgRNAs) targeting the coding region of the first exon of *IDO1* were cloned into PX458 (plasmid 48138, Addgene). These sgRNA-containing plasmids were transfected into HeLa cells using Lipofectamine 3000 (L3000008, Thermo Fisher Scientific) along with a synthetic repair plasmid consisting of mCherry coupled to the SV40 polyadenylation sequence and ~500 nt flanking sequence on either side of exon 1 of *IDO1*. Successful homologous recombination replaced the part of the first coding exon of *IDO1* with mCherry. Following sorting for GFP^+^ cells, single cell cloning in 96-well plates was performed to isolate individual clones, which were next expanded and selected based on mCherry expression and immunoblot analysis upon IFNγ stimulation. The following oligonucleotides were used:

IDO1_1) Forward 5’ ---- 3’ CACCGCACACGCTATGGAAAACTCC

IDO1_1) Reverse 5’ ---- 3’ AAACGGAGTTTTCCATAGCGTGTG

IDO1_2) Forward 5’ ---- 3’CACCGAGCCCACTTCTTCATCAATA

IDO1_2) Reverse 5’ ---- 3’ AAACTATTGATGAAGAAGTGGGCT

IDO1_3) Forward 5’ ---- 3’CACCGTACCATATTGATGAAGAAGT

IDO1_3) Reverse 5’ ---- 3’ AAACACTTCTTCATCAATATGGTA

To generate AHR and NRF2 deficiencies in HeLa cells, sgRNA sequences were designed using standard tools or taken from the literature (Michlits et al., 2020) and cloned into the PX458 CRISPR vector. HeLa cells were transfected with sgRNA-containing plasmids using Lipofectamine 3000. Single cell cloning was performed directly following sorting for GFP^+^ cells in 96-well plates to isolate individual ko clones, which were then selected based on the target protein expression and sequencing. NRF2 ko was further confirmed by western blot analysis after 6 hr treatment with 10 μM MG132 (474790, Millipore).

The following oligonucleotides were used:

AHR_1) Forward 5’ ---- 3’ CACCGGGCAGCAGGCTAGCCAAA

AHR_1) Reverse 5’ ---- 3’AAACTTTGGCTAGCCTGCTGCCCC

NRF2_1) Forward 5’ ---- 3’CACCGGACAAGAACAACTCCAAA

NRF2_1) Reverse 5’ ---- 3’AAACTTTGGAGTTGTTCTTGTCC

#### siRNA and plasmids

HeLa cells were seeded at 2 × 10^5^ cells/well in six well plates. After 24 hr incubation at 37°C, the cells were transfected with 10 nM of SLC7A11 siRNA (4390824, s24289, Invitrogen), scrambled siRNA (4390843, Invitrogen) or ON-TARGETplus Human KYNU siRNA (L-009867-00-0005, Horizon Discovery) using Lipofectamine RNAiMAX Transfection Reagent (13778150, Invitrogen) according to the manufacturer’s instructions. Cells were re-plated 6 hr after transfection for the ferroptosis assay and western blot analysis. SLC7A11 (NM_014331, Origene) and KYNu 3x HA plasmid DNA were transiently transfected into HeLa cells using Lipofectamine 3000 Transfection reagent (L3000008, Invitrogen) for 48 hr before proceeding to specific treatments.

#### Co-culture assay

Wt or IDO1 ko HeLa cells were treated for 3hr at 37°C with 10 µg/ml of Mitomycin C (M4287, Sigma-Aldrich), seeded at 5 × 10^4^ cells/well in 96 well plates and co-cultured with T-cells in a 1:2 ratio, respectively, in presence of 2 µl/well of CD3/CD28 dynabeads (111.61D, Thermo Fisher Scientific). Primary human CD8^+^ T cells were a gift from Prof. Vincenzo Bronte (University of Verona). For the quantification of T cells proliferation primary human CD8^+^ T cells were resuspended at a final concentration of 10^7^ cells/ml in PBS and stained with 2.5 μM as final working concentration of CellTrace™ CFSE (C1157, Invitrogen), followed by 5 min of incubation at 37 °C, protected from light. Cells were then washed and resuspended in culture medium and added to the HeLa cells for 4 days. CellTrace signal of gated lymphocytes was acquired by a Fortessa flow cytometer and analyzed by FlowJo software.

When needed, the supernatant of IFNγ-stimulated HeLa cells (48 hr) was fractionated with Amicon-Ultra 10 kDa columns (UFC501024, Merck).

#### Targeted metabolomics

Samples for metabolomics analysis were prepared after 48 hours of co-culture; after centrifugation, the supernatants of the co-colture were shock frozen with liquid nitrogen, and stored at −80 °C. HeLa cells (10^7^ cells) were centrifuged at low speed (5 min, 800 g, 4°C) to gently pellet the cells. Cell pellets were extracted using a pre-cooled MeOH:ACN:H_2_O (2:2:1, v/v) solvent mixture (-20°C). A volume of 1 mL of the cold solvent mixture was added to each pellet, vortexed for 30 s, and incubated in liquid nitrogen for 1 min. The samples were then allowed to thaw at room temperature and sonicated for 10 min in a water bath sonicator (4°C). The cycle of cell lysis in liquid nitrogen combined with vortex and sonication was repeated (in total two times & using the same suspension without adding fresh solvent mixture again). To precipitate proteins, the samples were incubated for 1 hr at −20 °C, followed by a 15 min centrifugation at 13,000 rpm at 4 °C. After centrifugation, the supernatants were snap frozen with liquid nitrogen, and stored at −80 °C.

For the quantification of TRP, KYN and phenylalanine reversed phase chromatography has been used. Briefly, 1 μl of the metabolite extracts has been separated on a Kinetex (Phenomenex) C18 column (100 Å, 150 x 2.1 mm) connected with the respective guard column, employing a 10-minute-long linear gradient from 99% A (1 % acetonitrile, 0.1 % formic acid in water) to 80% B (0.1 % formic acid in acetonitrile) at a flow rate of 100 µl/min. On-line tandem mass spectrometry (LC-MS/MS) was performed by employing the selected reaction monitoring (SRM) mode of a TSQ Vantage mass spectrometer (Thermo Fisher Scientific), using the transitions *m/z* 205 → *m/z* 188, CE=10 (TRP), *m/z* 209 → *m/z* 192, CE=10 (KYN) and *m/z* 166 → *m/z* 103, CE=28 (phenylalanine) in the positive ion mode. For the analysis of deuterium labelled kynurenine, the transition *m/z* 215.1 → *m/z* 198, CE=10 (D6 kynurenine, aromatic ring-D4, 3,3-D2) was used.

In an independent analysis, 1 µl of the samples were injected onto a SeQuant ZIC-pHILIC HPLC column (Merck, 100 x 2.1 mm; 5 µm) and the respective guard column, operated at a flow rate of 100 µl/min. The HPLC (Ultimate 3000 HPLC system; Dionex, Thermo Fisher Scientific) was directly coupled via electrospray ionization to a TSQ Quantiva mass spectrometer (Thermo Fisher Scientific). A linear gradient (A: 95% acetonitrile 5%, 10 mM aqueous ammonium acetate; B: 5 mM aqueous ammonium bicarbonate) starting with 15% B and ramping up to 60% B in 9 minutes was used for separation. The following SRM transitions were used for quantitation ion the negative ion mode: *m/z* 124 → *m/z* 80, CE=20 (taurine), *m/z* 188 → *m/z* 144, CE=12 (Kynurenic acid), *m/z* 152 → *m/z* 108 CE=14 (3-hydroxy anthranilic acid), *m/z* 662 → *m/z* 540, CE=12 (NAD) and *m/z* 664 → *m/z* 408, CE=29 (NADH).

Data interpretation was performed using TraceFinder (Thermo Fisher Scientific). Authentic metabolite standards were used for determining collision energies and retention times and for validating experimental retention times by standard addition. Ion intensities for each metabolite have been calculated from two replicates of the average ion count area values of each transition.

#### Ferroptosis experiments

0.01-0.02 × 10^4^ cells were seeded in 48-well plates and ferroptosis was induced 24 hr later with either 10 μM erastin (S7242, Selleckchem) or 1 μM RSL3 (S8155, Selleckchem). When indicated, 2 μM ferrostatin-1 (SML0583, Sigma-Aldrich) was used to protect from ferroptotic cell death. Cells were monitored by live phase- contrast microscopy using the IncuCyte^®^ S3 Live Cell Analysis System (Sartorius, USA) with the 10X objective, taking nine images per replicate every 2 hr for 48 hr. Cell death was monitored over time using CellTox Green (G8731, Promega) counting for green object count.

#### Cell death analysis by flow cytometry

Ferroptosis was induced using 10 μM erastin (S7242, Selleckchem) or 1 μM RSL3 (S8155, Selleckchem) in the presence of 200 μM of the metabolites for 24 hr. For the staining, cells were harvested, washed one time in PBS and stained with 2 μl of 7-AAD (559925, BD Biosciences) and 1 μl of Annexin-V-ACP (550474, BD Biosciences) in 100 μl Annexin-V binding buffer (550475, BD Biosciences). After 15 min, cells were recorded on the LSRII with the FACS Diva 6.1.1 software (BD Biosciences).

#### DPPH scavenging assay

Radical scavenging activity was analyzed using the stable free radical 1,1-diphenyl-2-picrylhydrazyl (DPPH) (D9132, Sigma). All compounds were added to a 200 µM DPPH solution in pure methanol. After 10 min incubation, the absorbance was measured at 517 nm. The decrease in absorbance due to radical scavenging was calculated relative to the H_2_O control.

#### Measurement of ROS production

Cells were treated with erastin to induce ferroptosis for 8 hr in the presence of KP metabolites, 30 minutes before the end of the experiment 10 μM of H_2_DCFDA (H_2_DCFDA (H2-DCF, DCF), D399 Thermo Fisher Scientific) was added. The MFI in the cells was measured by flow cytometry; due to difference in the autofluorescence of the KP metabolites, we subtracted the MFI of the same cells treated with the KP metabolites but without erastin (LSR Fortessa; BD). The results were analyzed by FlowJo 10 software.

#### Measurement of lipid peroxidation

Cells were treated with erastin to induce ferroptosis for 8 hr in the presence of KP metabolites. Lipid peroxidation was assessed using C11-BODIPY 581/591 (Molecular Probes/Life Technologies) according to the manufacturer’s instructions. The MFI in the cells was measured by flow cytometry; due to difference in the autofluorescence of the KP metabolites, we subtracted the MFI of the same cells treated with the KP metabolites but without erastin (LSR Fortessa; BD). The results were analyzed by FlowJo 10 software.

#### KYN uptake assay

200 μM KYN (K8625, Sigma Aldrich) was added to the cells and incubated for the indicated times at 37 °C. The uptake was stopped washing the cells one time with PBS 1X then trypsinizing the cells and adding 4% PFA for 10 min at RT. KYN uptake was monitored by flow cytometry analyzing the mean fluorescence intensity (MFI) using the 405 nm laser and 450/50 BP filter (Sinclair et al., 2018). The results were then analyzed by FlowJo 10 software.

#### Cell based transport assays

HeLa cells were maintained under 5% CO2 at 37 °C in RPMI 1640 medium supplemented with 2 mM L-glutamine and 10 % foetal bovine serum. 2 x 10^5^ cells (per well) were seeded into 12 well plates and 24 hr later, transfected using lipofectamine 2000 with equal amounts of SLC7A11/SLC3A2 plasmids for 36 hr. Cells were washed twice with 1 ml of PBS before application of 0.4 ml PBS containing ^14^C-cystine (^14^C-CYS, 0.3 μM) and 1 mM competing substrate. After 10 minutes the assay buffer was removed, and the cells quickly washed twice with 0.5 ml assay buffer with no cystine. Cells were removed using trypsin (0.1% in PBS for 2 minutes) and placed in a scintillation vial containing 100 µl 1M NaOH and lysed for 5 minutes prior to the addition of scintillation fluid. The amount of CYS taken up by cells in the absence of a competitor was set as 100 % transport. Experiments were performed a minimal of six times to generate an overall mean and SDs.

#### Cystine-FITC uptake assay

Cells were treated with 200 μM KYN, 3HK and HAA for 24 hr and 100 μM BioTracker Cystine-FITC (CT047, Merck) was added to the cells in a Cystine-free medium. Cells were incubated for 5 min at 37 °C. Subsequently, cells were washed with PBS 1x and re-suspended in FACS buffer. The MFI of the cells was determined by flow cytometry (LSR FortessaTM; BD). The results were analyzed by FlowJo 10 software.

#### RNAseq

Wt or IDO1 ko HeLa cells were treated in triplicates for 24 hr with 10ng/ml IFNγ, 1 µM epacadostat, 1 µM BMS986205 and 100 µM of indoximod. RNA was extracted using Qiagen RNeasy Mini kits (Qiagen) according to the manufacturer’s protocol. mRNA sequencing libraries were prepared with 1 µg of total RNA of each sample using the NEBNext Ultra II Directional RNA Library Prep Kit for Illumina (E7765, NEB) with NEBNext Poly(A) mRNA Magnetic Isolation Module (E7490, NEB), according to standard manufacturer’s protocol. Total RNA and the final library quality controls were performed using Qubit Flex Fluorometer (Q33327, Thermo Fisher Scientific) and 2100 Bioanalyzer Instrument (G2939BA, Agilent) before and after library preparation. Paired-end sequencing was performed on Illumina NextSeq 500 (2 × 43 bp reads). The samples were multiplexed and sequenced on one High Output Kit v2.5 to reduce a batch effect. BCL raw data was converted to FASTQ data and demultiplexed by bcl2fastq Conversion Software (Illumina). BAM and bigwig files are generated by STAR alignment and file conversion scripts – bam2wig and wigToBigWig.

After checking the quality of the samples (FastQC, v.0.11.7: https://www.bioinformatics.babraham.ac.uk/projects/fastqc/), the files were mapped to the human genome (Genome build GRCh38) downloaded from Ensembl using the star aligner v. 2.7.4a, (Dobin et al., 2013). The mapped files were then quantified on a gene level based on the ensembl annotations, using the featureCounts (Liao et al., 2014) (v. 2.0.1) tool from the SubRead package (Liao et al., 2013)(v. 2.0.1). Using the DESeq2 package (R 4.0.3, DESeq version 1.30.1)(Anders and Huber, 2010) the count data was normalized by the size factor to estimate the effective library size. A filtering step of removing genes with less than 10 reads in at least three samples was used. This followed by the calculation of gene dispersion across all samples. The analysis of two different conditions against each other resulted in a list of differentially expressed genes for each comparison. Genes with an adjusted p-value of <=0.05 were then considered to be differentially expressed for downstream analysis.

#### Immunoblotting

Lysates were prepared on ice in RIPA buffer (ab156034, Abcam) containing protease and phosphatase inhibitors (78444; Thermo Fisher). Proteins were separated on Tris-HCl gradient gels (5678084-5678085; BioRad) and transferred to 0.2 µM nitrocellulose (N010600001, Amersham). Membranes were blocked in 3% BSA (A2153; Sigma) or 3% nonfat milk (T145.2; Carl Roth) in TBS 0.01% Tween 20 and probed with primary antibodies overnight at 4 °C. Membranes were washed and probed with secondary antibodies at a 1:10000 dilutions and developed using chemiluminescence reagents (SuperSignal™ West Pico PLUS Chemiluminescent Substrate, 34580, Thermo Fisher Scientific).

#### Immunofluorescence (IF)

To determine the intracellular accumulation of KYN, 48 hr after transfection with SLC7A11 and the control plasmid, cells were plated on µ-Slide 8 Well Glass Bottom coverslips (80827, ibidi), treated with 200 µM KYN, washed one time with PBS and fixed in 4% formaldehyde for 15 minutes at RT. After washing 3 times with PBS1x, cells were blocked and permeabilized with 3% BSA + 0.2% Triton X-100 in PBS x for 90 minutes at RT. To detect the intracellular signal, a mouse monoclonal anti-L-KYN antibody (3D4-F2, IS003, Immusmol) was used in PBS 1x containing 3% BSA overnight at 4°C. A secondary goat anti-mouse IgG (H+L) antibody (Alexa Fluor 488, 1:1000; A11029; Invitrogen) was then used in 3% BSA in PBS 1x solution for 1 hr at RT followed by nuclei staining with DAPI (1:20000, D9542 Sigma Aldrich) in PBS 1x for 15 minutes at RT.

### Quantification And Statistical Analysis

The significance of differences in the experimental data were determined using GraphPad Prism software. All data involving statistics are presented as mean ± SD. The number of replicates and the statistical test used are described in the figure legends. Molecular surface representations were performed using PyMol.

## Supplementary Material

Supplementary Information

Table S1 related to Fig. 1 TRP and KYN metabolites

Video S1 related to Fig. 1. T cell-driven IDO1 reporter activity

## Data Availability

The RNA-seq dataset generated during this study is deposited at Gene Expression Omnibus (GEO) and is publicly available as the date of publication. Accession numbers is listed in Key resources table. Original western blot, microscopy images and metabolomic data have been deposited at the Mendeley https://data.mendeley.com/datasets/ywhjnx4mst/2. This paper does not report original code. Any additional information required to reanalyze the data reported in this paper is available from the lead contact upon request.
