# Supplementary material for: Kynurenine importation by SLC7A11 propagates anti-ferroptotic signaling
Source: Mol Cell. Author manuscript; Available in PMC 2025 Jul 20. (PMC7617904; doi:10.1016/j.molcel.2022.02.007)
Supplement: Supplementary Information [file EMS206716-supplement-Supplementary_Information.docx]

**Supplemental information**

**Kynurenine importation by SLC7A11 propagates anti-ferroptotic signaling**

Alessandra Fiore, Leonie Zeitler, Marion Russier, Annette Groß, Maria-Kathrin Hiller, Joanne L. Parker, Luca Stier, Thomas Köcher, Simon Newstead and Peter J. Murray

**Supplementary figure 1. Accurate monitoring of IDO1 expression and activity in tumor cells. Related to Figure 1.**(A) Schematic depicting the construction of the IDO1-mCherry reporter cell line.
(B) Immunoblot analysis of IDO1 expression in wt and IDO1 ko controls and IFNγ-treated conditions.
(C) Quantification of IDO1 expression with IDO1-mCherry reporter cells by tracking mCherry by live imaging upon IFNγ-treatment.
(D) Representative microscopy images IDO1-mCherry reporter cells from the experiment in (C).
(E) Normalized intensity (area) for TRP, KYN, KYNA, 3-Hydroxyanthranilic acid (HAA), NADH and NAD^+^ detected by LC-MS-based targeted metabolomics in the cell lysates after 48 hr of co-culture between wt or IDO1 ko HeLa cells with CD8^+^ lymphocytes.
(F) Schematic depicting the experimental approach used in G and H.
(G, H) 24 hr of pre-conditioning of wt (B) of IDO1 ko (C) HeLa cells with the supernatant from 48 hr untreated or IFNγ –treated HeLa cells after size-filtration protects from erastin- and RSL3- induced ferroptosis (left and right, respectively). Cell death was monitored after 48 hr using CellTox, counting for green objects normalized to cell confluence. n=3 biological replicates, bars are SDs *p < 0.05 two-tailed Student’s t test for pairwise comparisons in (E, G, H). *p < 0.05, **p < 0.01, ***p < 0.001

**
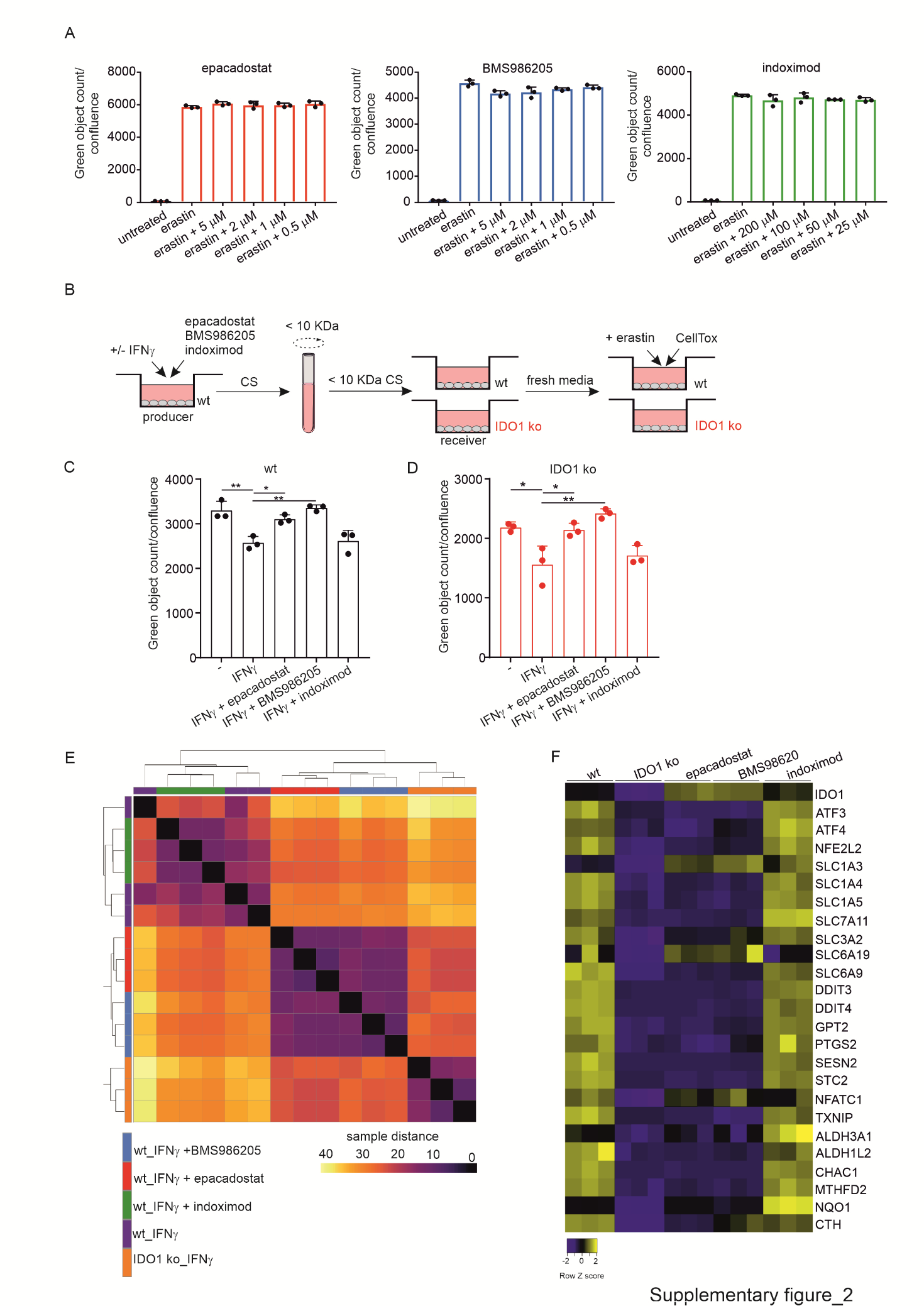
**

**Supplementary figure 2. Assessment of IDO1i specificity. Related to Figure 1 and 2**(A) Quantification of erastin-induced cell death in Hela cells in the presence of increasing doses of epacadostat, BMS-986205 and indoximod. Cell death was monitored after 48 hr using CellTox, counting for green objects normalized to cell confluence.
(B) Schematic depicting the experimental approach used in B and C.
(C, D) Quantification of erastin-induced cell death in wt (C) or IDO1 ko (D) HeLa cells pre-conditioned for 24 hr with supernatant (<10 kDa size filtrated) from 48 hr untreated or IFNγ –treated HeLa cells, in the presence of 2 µM epacadostat, 2 µM BMS-986205 and 100 µM of indoximod.
A, C and D n=3 biological replicates, bars are SDs *p < 0.05, **p < 0.01, ***p < 0.001 for multiple comparisons calculated using one-way ANOVA with Tukey’s HSD test (C, D).
(E) HeLa cells were treated for 24 hr with IFNγ in the presence of 2 µM BMS-986205, 2 µM epacadostat and 100 µM of indoximod and RNAseq analysis was used to quantify gene expression changes of IFNγ –treated wt versus IDO1 ko HeLa cells. (n=3 biological replicates). Heatmap of the variance-stabilizing transformation. The transformation calculates the within-group variability and so clustered samples with similar behavior closer to each other. The heatmap plots the distances between the samples. The closer (e.g. more similar) they are the lower the value.
(F) Heat map of relative gene expression of selected genes from (E) with adjusted p-value <0.05.

**
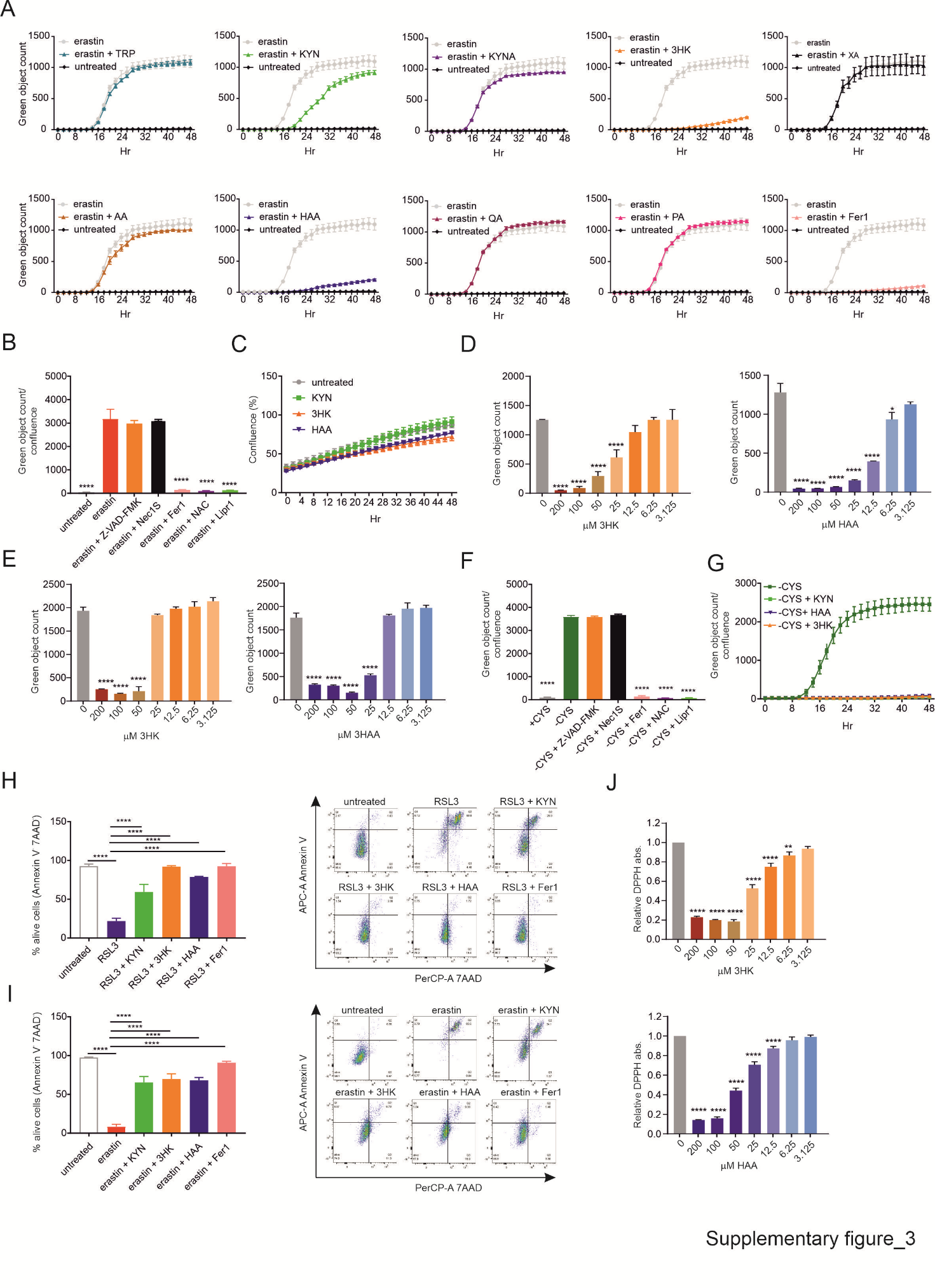
**

**Supplementary figure 3. KYN metabolites protect against ferroptosis by radical scavenging. Related to Figure 3.**(A) Over time quantification of erastin-induced cell death in HeLa cells treated with 10 µM of erastin in the presence of 200 µM of TRP, KYN, KYNA, 3HK, xanthurenic acid (XA), anthranilic acid (AA), HAA, quinolate (QA), and picolinate (PA). 2 µM of Fer-1 was used as a control to block ferroptosis.
(B) HeLa cells concurrently treated for 48 hr with erastin in the presence of 20 µM Z-VAD-FMK, 10 µM Necrostatin1S (Nec1S), 2 µM Ferrostatin1 (Fer1), 1 mM NAC or 2 µM Liproxtatin1(Lipr1).
(C) Quantification of cell confluence over time of HeLa cells grown in the presence of 200 µM of KYN, 3HK and HAA.
(D, E) HeLa cells were concurrently treated for 48 hr with erastin (D) or RSL3 (E) in the presence of decreasing doses of 3HK (left) and HAA (right) starting from 200 µM.
(F) HeLa cells deprived of CYS for 48 hr in the presence of 20 µM Z-VAD-FMK, 10 µM Necrostatin1S (Nec1S), 2 µM Ferrostatin1 (Fer1), 1 mM NAC or 2 µM Liproxtatin1(Lipr1).
(G) HeLa cells were deprived of cysteine for 48 hr in the presence of 200 µM of KYN, 3HK and HAA.
Cell death was monitored over time using CellTox, counting for green objects normalized to cell confluence.
(H, I) Flow cytometry analysis of the anti-ferroptotic activity of KYN, 3HK and HAA towards RSL3 (G) and erastin- (H) induced ferroptosis. Cell death was quantified by 7-AAD and Annexin-V staining (Representative plots on the left). n = 3 biological replicates, bars are SDs.
(J) Titration of cell-free scavenging activity of 3HK (up) and HAA (down) starting from 200 µM.
(A-I) n = 3 biological replicates, (J) n = 4 technical replicates; bars are SDs **p < 0.01, ***p < 0.001, ****p < 0.0001 for multiple comparisons calculated using one-way ANOVA with Dunnett’s test (B, D, E, F, H, I, J).

**
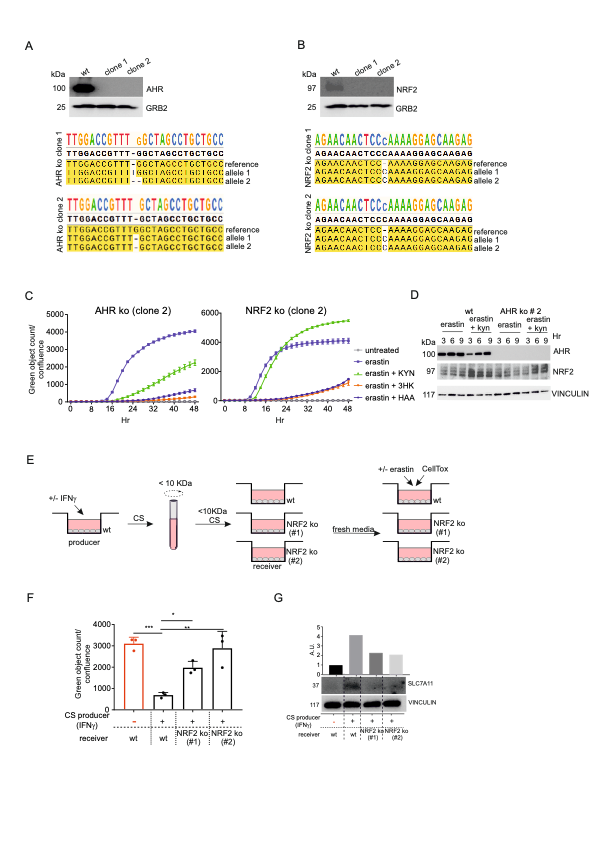
**

**Supplementary figure 4. KYN metabolites protect against ferroptosis through NRF2. Related to Figure 5**(A) Immunoblot validation and sequence alignment of AHR ko clones in HeLa cells.
(B) Immunoblot validation and sequence alignment of NRF2 ko clones in HeLa cells. For protein validation of NRF2 ko cells were treated for 6 hr with 10 μM MG132.
(C) AHR ko (left) or NRF2 ko (right) HeLa cells were concurrently treated for 48 hr with erastin in the presence of 200 µM KYN, 3HK and HAA. Cell death was monitored over time using CellTox, counting for green objects normalized to cell confluence (n = 3 biological replicates, bars are SDs). 
(D) Immunoblot validation of NRF2 and AHR induction in wt and AHR ko HeLa cells.
(E) Schematic depicting the experimental approach used in F and G. CS, conditioned supernatant.
(F) Quantification of erastin-induced cell death in wt or NRF2 ko HeLa cells pre-conditioned for 24 hr with supernatant (<10 kDa size filtrated) from 48 hr untreated or IFNγ –treated HeLa cells. Cell death was monitored at 48 hr using CellTox, counting for green objects normalized to cell confluence.
(G) Immunoblot analysis and quantification of the protein blots relative to the vinculin loading control of SLC7A11 in wt and NRF2 ko HeLa cells treated for 24 hr with the supernatant (<10 kDa size filtrated) from 48 hr untreated or IFNγ –treated HeLa cells. n = 3 biological replicates, bars are SDs. **p < 0.01, ****p < 0.0001 for multiple comparisons calculated using one-way ANOVA with with Tukey’s HSD test.

**Supplementary figure 5. Human SLC7A11 displays a strong sequence identity to the KYN transporters SLC7A5 and mediates KYN transport in cancer cells. Related to Figure 6**(A) Multiple sequence alignment of human LAT1 (SLC7A5), LAT2 (SLC7A8) and xCT (SLC7A11).
(B) HeLa cells were transfected with SLC7A11 siRNA (siCTRL as negative control). 48 hr after transfection KYN uptake was quantified by flow cytometry upon 5 min of treatment with 45 µM of KYN.
(C) Quantification of D6-KYN uptake after 1 and 10 min of treatment with 45 µM of D6-KYN into transfected cells as in B.
(D-E) SKOV3, (F,G) PT45, (H, I) U2OS and (J,K) HT1080 cells were transfected with SLC7A11 siRNA (siCTRL as negative control). 48 hr after transfection SLC7A11 knock down was confirmed by immunoblot (D, F, H, J) and KYN uptake was quantified by flow cytometry upon 5 min of treatment with 200 µM of KYN ( E, G, I, K).
(L) Flow cytometry evaluation of KYN uptake after 5 min of treatment with 200 µM of KYN in the presence of 50 µm of JPH203, 250 µM of Sulfasalazine (SAS), 10 µm of erastin or 5 mM of BCH.
(M,N) HeLa cells were transfected with SLC7A11-overxpressing plasmid (empty vector was used as negative control). 48 hr after transfection SLC7A11 overexpression was confirmed by immunoblot (M) and KYN uptake was quantified by flow cytometry upon 5 min of treatment with 200 µM of KYN (N).
n = 3 biological replicates, bars are SDs. *p < 0.05, **p < 0.01, ***p < 0.001, ****p < 0.0001 unpaired t-test for B, C, one-way ANOVA with Tukey’s HSD test (E, G, I, K, N) and Dunnett’s test for J.

**Supplementary figure 6. SLC7A11 mediates KYN transport. Related to Figure 6**(A) Immunofluorescence of KYN accumulation in CTRL HeLa cells and HeLa cells transfected with SLC7A11-overexpressing plasmid after 5, 15 or 60 minutes of treatment with 200 µM KYN (representative images of one of three independent transfection experiments).
(B) Flow cytometry analysis of cystine-FITC (CYS-FITC) uptake in HeLa cells. Cells were pre-treated for 24 hr with 200 µM KYN, 3HK and HAA and incubated for 5 minutes with FITC-labelled cystine in cystine –depleted medium (n = 3 biological replicates, bars are SDs).
(C) Flow cytometry evaluation of KYN uptake after 5 min of treatment with 45 µM of KYN and increasing concentration of CYS compared to normal medium
(D) Quantification of D6-KYN uptake after 5 min of treatment with 45 µM of D6-KYN and increasing concentration of CYS into transfected cells as in B.
(E) Immunoblot of HeLa cells concurrently treated with 200 µM KYN for 12 or 24 hr and 500 nM of GCN2i.
 (n = 3 biological replicates, bars are SDs). *p < 0.05, **p < 0.01, ***p < 0.001, ****p < 0.0001 for multiple comparisons calculated using one-way ANOVA with Dunnett’s test for B and C and unpaired t-test for D.
(D) Immunoblot of HeLa cells concurrently treated with 200 µM KYN for 12 or 24 hr and 500 nM of GCN2i.
